# Charge‐Polarized Selenium Vacancy in Nickel Diselenide Enabling Efficient and Stable Electrocatalytic Conversion of Oxygen to Hydrogen Peroxide

**DOI:** 10.1002/advs.202205347

**Published:** 2022-12-07

**Authors:** Yingming Wang, Hui Huang, Jie Wu, Hongyuan Yang, Zhenhui Kang, Yang Liu, Zhaowu Wang, Prashanth W. Menezes, Ziliang Chen

**Affiliations:** ^1^ Institute of Functional Nano & Soft Materials (FUNSOM) Jiangsu Key Laboratory for Carbon‐Based Functional Materials & Devices Soochow University 199 Ren'ai Road Suzhou Jiangsu 215123 P. R. China; ^2^ Department of Chemistry: Metalorganics and Inorganic Materials Technische Universität Berlin Straße des 17 Juni 135, Sekr. C2 10623 Berlin Germany; ^3^ School of Physics and Engineering Henan University of Science and Technology Luoyang 471023 P. R. China; ^4^ Material Chemistry Group for Thin Film Catalysis ‐ CatLab Helmholtz‐Zentrum Berlin für Materialien und Energie Albert‐Einstein‐Str. 15 12489 Berlin Germany

**Keywords:** anion vacancy, charge polarization, hydrogen peroxide, oxygen reduction reaction, transition metal chalcogenide

## Abstract

Vacancy engineering is deemed as one of the powerful protocols to tune the catalytic activity of electrocatalysts. Herein, Se‐vacancy with charge polarization is created in the NiSe_2_ structure (NiSe_2_‐*V*_Se_) via a sequential phase conversion strategy. By a combined analysis of the Rietveld method, transient photovoltage spectra (TPV), in situ Raman and density functional theory (DFT) calculation, it is unequivocally discovered that the presence of charge‐polarized Se‐vacancy is beneficial for stabilizing the structure, decreasing the electron transfer kinetics, as well as optimizing the free adsorption energy of reaction intermediate during two‐electron oxygen reduction reaction (2e^−^ ORR). Benefiting from these merits, the as‐prepared NiSe_2_‐*V*_Se_ delivered the highest selectivity of 96% toward H_2_O_2_ in alkaline media, together with a selectivity higher than 90% over the wide potential range from 0.25 to 0.55 V, ranking it in the top level among the previously reported transition metal‐based electrocatalysts. Most notably, it also displayed admirable stability with only a slight selectivity decay after 5000 cycles of accelerated degradation test (ADT).

## Graphical abstract

Nickel diselenide with negative charge‐polarized Se vacancy (NiSe_2_‐*V*_se_) has been developed for the two‐electron oxygen reduction reaction (2e^−^ ORR), which delivered remarkable activity, selectivity, and stability. Furthermore, the presence of negatively charge‐polarized Se vacancy effectively retards the interfacial charge transfer and optimizes the adsorption free energy toward *OOH intermediate.

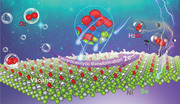

## 1 Introduction

Hydrogen peroxide (H_2_O_2_) is one of the most valuable oxidants and has found extensive applications in chemical and medical industries, as well as in wastewater treatment and textile bleaching.^[^ ^1^, ^2^, ^3^ ^]^ Currently, commercial H_2_O_2_ is widely produced via the energy‐intensive anthraquinone process during which environmental pollution, transport, and handling risks are involved.^[^ ^4^, ^5^, ^6^, ^7^ ^]^ Therefore, for a sustainable future, it is essential to develop an energy‐efficient and eco‐friendly strategy for the synthesis of H_2_O_2_ that must operate on‐site even larger or on small scales. In this respect, electrocatalytic 2e^−^ oxygen reduction reaction (2e^−^ ORR) has been deemed as the most promising alternative approach as it can realize the green and distributed on‐demand H_2_O_2_ generation under ambient conditions.^[^ ^8^, ^9^, ^10^ ^]^ However, the favorable thermodynamics to generate water molecules via the 4e^−^ pathway inevitably reduces the capability for H_2_O_2_ generation during ORR. To this end, it is of paramount importance to search for an efficient and selective electrocatalyst to reduce O_2_ to H_2_O_2_ rather than H_2_O.

Over the last few years, many efforts have been made to develop non‐noble metal‐based alloys,^[^ ^11^, ^12^, ^13^, ^14^ ^]^ single metal atom,^[^ ^15^, ^16^, ^17^ ^]^ and carbon‐based materials^[^ ^18^, ^19^, ^20^, ^21^ ^]^ as advanced 2e^−^ ORR electrocatalysts. Despite the great progress that has been achieved, the design concept and catalytic performance for 2e^−^ ORR electrocatalysts still need to be further revolutionized.^[^ ^22^, ^23^ ^]^ As a pioneering work, Jiang et al. recently demonstrated the high potential of economic transition metal‐based selenides for electrosynthesis of H_2_O_2_, which is probably because they not only bear more metalloid characteristics than corresponding oxide and sulfide counterparts but are also tolerant under electrochemical conditions.^[^ ^24^, ^25^, ^26^ ^]^ Nevertheless, the reported selenide catalysts suffer from unsatisfactory intrinsic catalytic activity and selectivity toward 2e^−^ ORR and the optimized Cu_7.2_Se_4_ nanocrystals delivered only a 2e^−^ ORR selectivity of 90%.^[^ ^24^ ^]^ To address these issues and further improve their 2e^−^ ORR performance, vacancy engineering may be an effective approach as it can induce carrier migration, charge separation and variation of energy band structure, which indeed can effectively regulate the intrinsic electronic structure for catalyzing the surface reaction (e.g., tuning binding strength of active metal atom with O‐containing species).^[^ ^27^, ^28^, ^29^, ^30^, ^31^, ^32^, ^33^ ^]^ Based on such considerations, in 2020, Zou et al. introduced oxygen vacancy into Fe_2_O_3_ where more catalytic sites for O_2_ adsorption and protonation were achieved, and the cleavage of O—O bond in *OOH intermediate was inhibited to a large extent, thus leading to the 2e^−^ selectivity as high as 95%.^[^ ^34^ ^]^ Following the same principle, it has also been recently shown that the presence of oxygen vacancy could enable the high 2e^−^ selectivity up to ≈92% for TiO_2_ in alkaline media.^[^ ^35^ ^]^ Based on the results attained for oxides, it is highly anticipated that 2e^−^ ORR performance for metal selenides can be remarkably enhanced by creating the Se vacancy. Besides, the electron transfer kinetics on the catalyst's surface has a large impact on 2e^−^ ORR performance where usually a low charge transfer on the surface contributes to high selectivity toward 2e^−^ ORR.^[^ ^36^ ^]^ However, it still remains unknown whether and/or how the presence of vacancy affects the charge transfer behavior of transition metal‐based compounds during ORR.

Motivated by the above‐mentioned concerns, we deliberately synthesized a nanostructured NiSe_2_ catalyst with abundant Se vacancies (NiSe_2_‐*V*_Se_) through a sequential solvothermal–calcination–selenization–annealing treatment. By a combination of in/ex‐situ characterizations and density functional theory (DFT) calculations, it was unequivocally elucidated that the as‐prepared NiSe_2_‐*V*_Se_ exhibited the abnormal lattice expansion due to the negative charge‐polarized Se vacancy, endowing the catalyst with rich active sites, stable phase structure, decreased electron transfer rate as well as optimized adsorption free energy toward *OOH intermediate (ΔG_*OOH_). Benefiting from the above synergistic effect, the electrocatalyst displayed an excellent 2e^−^ ORR activity and selectivity, significantly superior to the pristine NiSe_2_, as well as most of the recently reported advanced 2e^−^ ORR electrocatalysts. Specifically, NiSe_2_‐*V*_Se_ delivered a notable H_2_O_2_ production selectivity, which was over 90% ranging from 0.25 to 0.55 V versus reversible hydrogen electrode (RHE), together with the highest selectivity of ≈96% at 0.45 V versus RHE. Moreover, such a catalyst was able to maintain its outstanding working efficiency for over 40 000s, as well as 5000 CV cycles.

## 2 Results and Discussion

**Figure 1:**
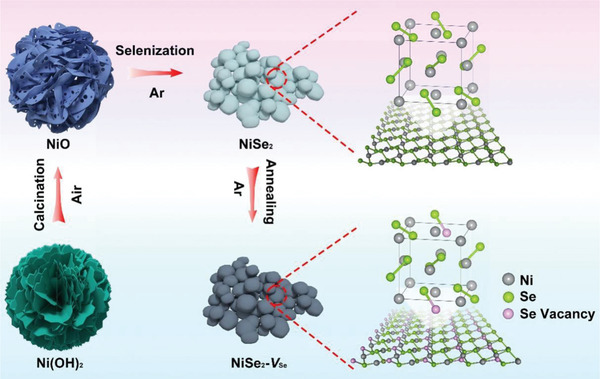
The synthetic scheme of NiSe_2_‐*V*_Se_ nanoparticles.

The synthesis procedure for NiSe_2_ with charge‐polarized Se‐vacancy (herein denoted as NiSe_2_‐*V*_Se_) is schematically displayed in **Figure** **1**. First of all, Ni(OH)_2_ nanoflowers assembled by nanosheets were prepared by one‐pot hydrothermal treatment. The Ni(OH)_2_ nanoflowers precursor was then calcined at 500 °C under air and during this process, Ni(OH)_2_ thermally decomposes into NiO nanoflowers with rough surfaces. The as‐obtained NiO was subjected to selenization using selenium powder at 500 °C under the N_2_ atmosphere yielding pristine NiSe_2_ nanochains. The morphology variation was probably due to the new phase crystallization and growth. Finally, the NiSe_2_ phase was thermally annealed at 350 °C for 30 min under N_2_ to create Se vacancies within the lattice structure and represented as NiSe_2_‐*V*_Se_.

**Figure 2:**
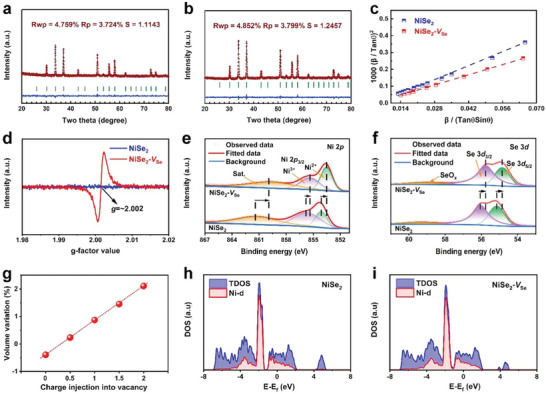
The Rietveld refinement of XRD patterns for a) NiSe_2_ and b) NiSe_2_‐*V*_Se_. The green line, red “+” symbol, blue line, and green vertical bars shown in figures (a) and (b) represents the observed data, fitted data, differentiation, and the positions of the diffraction peaks in the XRD patterns, respectively. c) Comparison of lattice strain in NiSe_2_ and NiSe_2_‐*V*_Se_. d) EPR spectra of NiSe_2_ and NiSe_2_‐*V*_Se_. High‐resolution XPS spectra of e) Ni 2p and f) Se 3d in NiSe_2_‐*V*_Se_. g) The dependence of the lattice parameter of NiSe_2_ on the variation of the charge state of the vacancies. The Ni metal *d*‐band center for h) NiSe_2_ phase and i) NiSe_2_‐*V*_Se_ phase (*E* − *E*_F_; relative to the Fermi level).

The phase conversion, as well as the purity of the materials during the synthesis, were first confirmed by measuring X‐ray diffraction (XRD). The XRD pattern clearly indicated that the pure Ni(OH)_2_ precursor could be successfully transformed into the NiSe_2_ phase (**Figure** **2**a,b, Figures S1 and S2, Supporting information). The structural differences for NiSe_2_ before and after annealing at 350  °C were further probed by the Rietveld refinement of their corresponding XRD patterns (Figure 2a,b). According to the refinement results (Table S1, Supporting Information), it was observed that although the samples both before and after annealing displayed the pure NiSe_2_ phase crystallizing in cubic structure (*Pa‐*3), the lattice volume of the latter was expanded by about 0.4% after annealing. Moreover, based on the Halder–Wagner method, the lattice strain after annealing was decreased (Figure 2c), meaning an increase in structural stability.^[^ ^36^ ^]^ The annealed NiSe_2_ showed a clear electron paramagnetic resonance (EPR) signal with a *g* value of 2.002 (Figure 2d), which was quite similar to the previous report,^[^ ^37^ ^]^ suggesting the existence of Se vacancies trapped with some electrons. Note that the generation of vacancy usually causes lattice shrinkage, such an abnormal lattice expansion observed in NiSe_2_ could be possibly due to the generation of negatively charged anion vacancy as EPR demonstrated, which was commonly observed in previous findings.^[^ ^38^, ^39^, ^40^, ^41^ ^]^ Besides, the surface chemical states of NiSe_2_ and NiSe_2_‐*V*_Se_ were investigated by X‐ray photoelectron spectroscopy (XPS) to further understand the effect of Se vacancy. Apparently, the high‐resolution Ni 2*p*_3/2_ XPS spectrum for NiSe_2_‐*V*_Se_ shifted to the lower binding energy as compared to that of pristine NiSe_2_, implying the decrease of the oxidation state of Ni in the presence of Se vacancies, which could be also confirmed by the lower atomic ratio of Ni^3+^/Ni^2+^ in NiSe_2_‐*V*_Se_ (0.818) than that in pure NiSe_2_ (1.915) (Figure 2e). In addition, the comparison of high‐resolution Se 3*d* XPS spectra for NiSe_2_ and NiSe_2_‐*V*_Se_ also revealed the decrease in the valence state of Se (Figure 2f). These valence variations could be attributed to the preservation of the structure to attain charge neutrality.^[^ ^42^, ^43^ ^]^ To further confirm the vacancy charge polarization‐induced lattice expansion, DFT calculations were performed. As shown in Figure 2g, the dependence of lattice parameters of NiSe_2_ on the charge state of Se vacancy showed that if no charge polarization occurred in Se vacancy, the lattice volume contracted, while when the Se vacancy was negatively charge‐polarized, the lattice volume linearly expanded. The Rietveld refinement demonstrated the 0.4% lattice expansion, corresponding to the negative charge less than −0.5 for Se vacancy in our model. Moreover, upon the presence of Se vacancies, the *d*‐band center of Ni species upshifted from −1.815 eV in pristine NiSe_2_ to −1.790 eV in NiSe_2_‐*V*_Se_, verifying again the electronic regulation by Se vacancy, which was regarded as beneficial for the adsorption of the active metal site toward reaction intermediates (Figure 2h,i).

**Figure 3:**
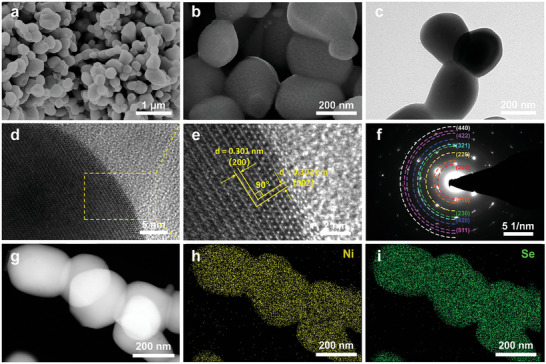
a,b) FESEM images, c) TEM image, d) high‐magnified and e) high‐resolution (HR)TEM images, as well as f) the corresponding SAED pattern of representative NiSe_2_‐*V*_Se_ particle. g) HAADF image of representative NiSe_2_‐*V*_Se_ particle and the corresponding EDX elemental mapping of h) Ni and i) Se.

Furthermore, we examined the microstructural features of NiSe_2_ and NiSe_2_‐*V*_Se_ through field‐emission scanning electron microscopic (FESEM) and transmission electron microscopic (TEM) characterizations. Interestingly, the nanosheet‐assembled nanoflower‐like morphology of the precursors (Ni(OH)_2_ and NiO) was converted into the nanochain connected by nanoparticles in pristine NiSe_2_, which also showed an average particle size of 200 nm (Figures S3–S5, Supporting Information). When it was further annealed, the obtained NiSe_2_‐*V*_Se_ could still inherit the morphology of pristine NiSe_2_ (**Figure** **3**a,b) very well, which was further demonstrated by the TEM findings. As shown in Figure 3c, NiSe_2_‐*V*_Se_ exhibited nanochain morphology and is consistent with results obtained from FESEM. Moreover, the corresponding high‐resolution TEM (HRTEM) image displayed lattice fringes with interspacing distances of 0.301 and 0.302 nm, which could directly be assigned to the (200) and (002) facets of NiSe_2_‐*V*_Se_, respectively (Figure 3d,e). Accordingly, these two equivalent crystal planes generated an included angle of 90° (Figure 3e). Furthermore, the selected area electron diffraction (SAED) pattern recorded from the particle region exhibited several bright diffraction rings/dots, which corresponded to the (200), (210), (220), (230) and (321) facets of NiSe_2_‐*V*_Se_ phase, respectively (Figure 3f). All of the above results achieved from TEM are in good agreement with the ones obtained from the Rietveld refinement of the XRD pattern for NiSe_2_‐*V*_Se_. In addition, the high angle annular dark field‐scanning TEM (HAADF‐STEM) pattern, as well as corresponding elemental mappings, further proved the homogeneous distribution of Ni and Se species within the whole NiSe_2_‐*V*_Se_ nanoparticle (Figure 3g–i). Note that negligible N species were detected in the as‐prepared NiSe_2_‐*V*_Se_ due to the thermal decomposition of hexamethylene tetramine (HMT) at high temperatures (Figure S6, Supporting Information). Similar results could also be observed in pristine NiSe_2_ (Figures S7 and S8, Supporting Information). It is important to note that the comparison of energy dispersive X‐ray (EDX) elemental mapping results suggested that the atomic ratio of Ni to Se in NiSe_2_‐*V*_Se_ (1:1.826) was significantly lower than that of the pristine NiSe_2_ (1:1.966), substantiating again the loss of Se species from host lattice after annealing (Figure S9, Supporting Information).

**Figure 4:**
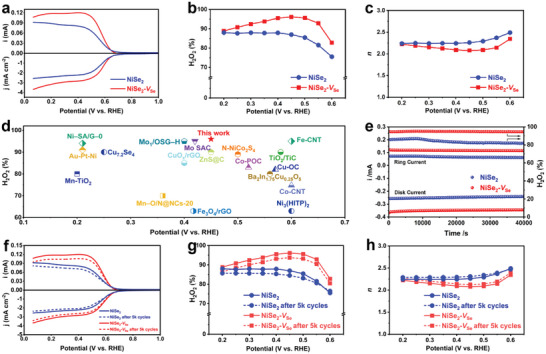
a) LSV curves of NiSe_2_ and NiSe_2_‐*V*_Se_ recorded at 1600 rpm with a scan rate of 10 mVs^−1^ (bottom part), together with the corresponding H_2_O_2_ current on the ring electrode (upper part). b) Selectivity of H_2_O_2_ and c) calculated electron transfer number (*n*) within the potential sweep. d) Comparison of 2e^−^ ORR activity of NiSe_2_‐*V*_Se_ with the previously reported Ni‐based and other TM‐based electrocatalysts in the alkaline media (for qualitative reference only). e) Stability tests of NiSe_2_ and NiSe_2_‐*V*_Se_ at a fixed disk potential of 0.4 V (versus RHE). f) LSV curves, g) selectivity of H_2_O_2_, and h) *n* value of NiSe_2_ and NiSe_2_‐*V*_Se_ after 5 k ADT cycles.

The electrocatalytic 2e^−^ ORR performance of NiSe_2_‐*V*_Se_ was evaluated via a rotating ring disk electrode (RRDE) (rotating rate: 1600 rpm) in O_2_‐saturated 0.1 m KOH, together with pristine NiSe_2_ for comparison.^[^ ^44^ ^]^ Prior to the performance test, the collection efficiency of the rotating ring disk electrode (RRDE) was determined and calculated as 0.37 (Figure S10, Supporting Information). The linear sweep voltammetry (LSV) curves were measured at a scan rate of 10 mV s^−1^, reflecting both the disk current density and ring current (**Figure** **4**a). As expected, NiSe_2_‐*V*_Se_ only required an onset potential of about 0.72 V versus RHE, which was quite close to the theoretical equilibrium value (0.7 V versus RHE).^[^ ^36^ ^]^ In addition, this catalyst even delivered 1 mA cm^−2^ disk current density at ≈0.603 V versus RHE, tremendously superior to the pristine NiSe_2_ catalyst (≈0.582 V versus RHE). Moreover, NiSe_2_‐*V*_Se_ could readily achieve a disk current density of 2.94 mA cm^−2^ at 0.3 V versus RHE, close to the theoretical diffusion‐limiting disk current density (3 mA cm^−2^),^[^ ^45^ ^]^ signifying the enhancement of 2e^−^ ORR activity by the introduction of Se vacancies. In addition to the activity, such a Se vacancy could also promote the 2e^−^ selectivity toward ORR. Specifically, NiSe_2_‐*V*_Se_ presented a high H_2_O_2_ selectivity that surpassed 90% in a wide potential window from 0.25 to 0.55 V versus RHE. The highest selectivity could reach up to 96% at 0.45 V versus RHE, which was much higher than that of pristine NiSe_2_ (87%) at the same potential (Figure 4b). Meanwhile, the positive role of Se vacancy in facilitating 2e^−^ ORR was further validated by the electron transfer number (*n*) during the ORR process. As shown in Figure 4c, NiSe_2_‐*V*_Se_ displayed an *n* value closer to 2 and it was much superior to that of NiSe_2_ in the measured range of 0.6 to 0.1 V (versus RHE). Notably, at 0.4–0.5 V (versus RHE), where the ring current more conclusively reflects the production of H_2_O_2_,^[^ ^46^, ^47^ ^]^ NiSe_2_‐*V*_Se_ displayed an *n* value of 2.07–2.10, suggesting that the presence of Se vacancy directed NiSe_2_ to undergo a near‐ideal 2e^−^ pathway. To the best of our knowledge, such an exceptional H_2_O_2_ selectivity toward 2e^−^ ORR enabled by NiSe_2_‐*V*_Se_ was even superior to previously documented all other Ni‐based and most low‐cost TM‐based electrocatalysts in the alkaline media (Figure 4d and Table S2, Supporting Information). To further investigate the dependence of Se vacancy on the annealing temperature as well as the related 2e^−^ ORR selectivity, NiSe_2_ was annealed at 250, 300, 400, and 450 °C (products are denoted as NiSe_2_‐*V*_Se_‐250, NiSe_2_‐*V*_Se_‐300, NiSe_2_‐*V*_Se_‐400, and NiSe_2_‐*V*_Se_‐450, respectively), and their corresponding XRD patterns (Figure S11, Supporting Information), EDX spectra (Figures S12 and S13, Supporting Information) as well as EPR spectra (Figure S14, Supporting Information) were provided. In conjunction with the characterization results for NiSe_2_‐*V*_Se_‐350 (i.e., NiSe_2_‐*V*_Se_), it could be known that all the compounds were pure NiSe_2_ phase and both the molar ratio of Ni to Se species and the EPR signal increased with the increase of the annealing temperature, meaning the increase of Se vacancies as the annealing temperature raises. Furthermore, the ORR performance in alkaline media for NiSe_2_‐*V*_Se_‐250, NiSe_2_‐*V*_Se_‐300, NiSe_2_‐*V*_Se_‐400, and NiSe_2_‐*V*_Se_‐450 was also tested and compared with that annealed under 350  °C (Figure S15, Supporting Information). From the results, it could be seen that the selectivity toward 2e^−^ ORR for NiSe_2_‐*V*_Se_‐250, NiSe_2_‐*V*_Se_‐300, NiSe_2_‐*V*_Se_‐400 and NiSe_2_‐*V*_Se_‐450 was inferior to NiSe_2_‐*V*_Se_ (350  °C), implying that the appropriate amount of Se vacancy was favorable for the enhancement of 2e^−^ ORR performance. On the other hand, the 2e^−^ ORR performance of NiSe_2_ and NiSe_2_‐*V*_Se_ in both neutral and acidic media was measured, and the results showed that the presence of Se vacancy was also conducive to improving the 2e^−^ ORR property (Figures S16 and S17, Supporting Information). It should be pointed out that their selectivity in both neutral and acidic media was lower than that in alkaline media, and further modifications such as phase engineering and interface coupling may be needed to improve their performance in the future.

Since stability is another key indicator to assess the performance of catalysts for alkaline electrosynthesis of H_2_O_2_, the chronoamperometry (CA) test at a potential of 0.4 V versus RHE was carried out for NiSe_2_‐*V*_Se_ (Figure 4e). Impressively, both the 2e^−^ ORR activity and selectivity basically remained unchanged for NiSe_2_‐*V*_Se_ during the 40 000s of duration. In addition, we also performed the 5000 cycles of accelerated degradation test (ADT) to further demonstrate the robust stability of NiSe_2_‐*V*_Se_. As shown in Figure 4f–h, only a slight activity and selectivity decay occurred for NiSe_2_‐*V*_Se_ even after 5000 ADT cycles. Moreover, the cycle performance of NiSe_2_‐*V*_Se_ was superior to that of NiSe_2_ (Figure 4g,h), and remarkably, the outstanding stability of NiSe_2_‐*V*_Se_ also exceeded nearly all reported Ni‐ and most other TM‐based alkaline 2e^−^ ORR catalysts (Table S2, Supporting Information). To further demonstrate the exceptional stability, the NiSe_2_‐*V*_Se_ was also cycled at the potential of 0.45 V (versus RHE) for 24 h. As displayed in Figure S18, Supporting Information, a little activity decay was observed for NiSe_2_‐*V*_Se_ after 24 h of the stability test, displaying its high durability.

Inspired by the activity, selectivity, and stability of NiSe_2_‐*V*_Se_ toward 2e^−^ ORR, the ability to produce H_2_O_2_ for NiSe_2_‐*V*_Se_ on a large scale was further explored. An H‐type electrolytic cell where carbon paper‐supported catalysts served as the working electrode, was assembled and tested in the O_2_‐saturated 0.1 m KOH electrolyte (Figure S19, Supporting Information). To determine the yield of the H_2_O_2_ product, the potassium permanganate titration method was employed. After 5000s chronoamperometry (CA) run at a fixed potential of 0.4 V versus RHE (corresponding to a current density of around 11.26 mA cm^−2^), a high H_2_O_2_ yield rate of about 720 mmol g^−1^_cat_ h^−1^ was attained by NiSe_2_‐*V*_Se_ (Figure S20, Supporting Information). In the meantime, an outstanding Faraday efficiency (FE) of about 95.73% could also be achieved (Figure S20, Supporting Information). Note that the slight destabilization of the evolved H_2_O_2_ under alkaline conditions and/or the occurrence of 4e^−^ ORR triggered by carbon paper probably led to a low FE value than the theoretical one.^[^ ^48^ ^]^

**Figure 5:**
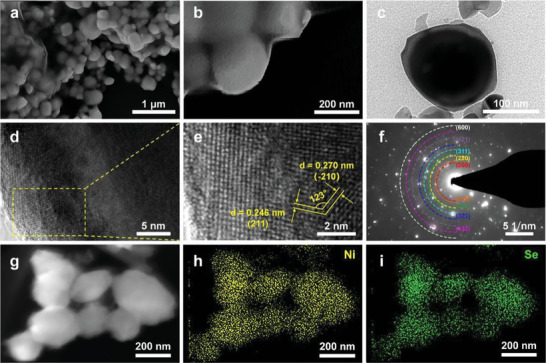
a,b) FESEM images, c) TEM image, d) high‐magnified and e) high‐resolution (HR)TEM images, as well as f) the corresponding SAED pattern of representative post‐ORR NiSe_2_‐*V*_Se_ particle. g) HAADF image of representative post‐ORR NiSe_2_‐*V*_Se_ particle and the corresponding EDX elemental mapping of h) Ni and i) Se.

To examine the morphology, phase, and microstructural stability of NiSe_2_‐*V*_Se_ after ORR CA, a series of post‐ORR characterizations including XRD, XPS, FESEM and TEM were conducted. It was evident that from XRD patterns, the phase structure was retained after ORR (Figure S21, Supporting Information). Moreover, the high‐resolution Ni 2*p* and Se 3*d* XPS spectra for post‐ORR NiSe_2_‐*V*_Se_ also exhibited quite similar behavior to those before ORR, illustrating that the chemical state of NiSe_2_‐*V*_Se_ marginally varied after ORR (Figures S22a,b, Supporting Information). The full XPS spectra and high‐resolution XPS spectra of oxygen species in NiSe_2_‐*V*_Se_ before and after ORR were also deconvoluted and are shown in Figure S23, Supporting Information. Only the oxygen signal from the adsorbed water could be identified in O 1s spectra which is consistent with the above results.^[^ ^49^ ^]^ Similarly, the surface morphology and microstructure of NiSe_2_‐*V*_Se_ after ORR were also thoroughly examined. As unveiled by FESEM images in **Figure** **5**a,b, the nanochains morphology was well‐preserved, which was further confirmed by its TEM image (Figure 5c). The high‐resolution TEM (HRTEM) image of such a particle is shown in Figure 5d and the selected magnified region is displayed in Figure 5e. As depicted in Figure 5e, the crystalline facets (211) and (−210) belonging to NiSe_2_‐*V*_Se_ could be clearly identified, which concurrently generated a contacted angle of 123°. The related selected area electron diffraction (SAED) pattern also presented the crystalline planes of (200), (210), (220), (221) and (311) of the NiSe_2_ phase, affirming the high stability of the crystalline phase during the electrocatalytic process (Figure 5f). Besides, the high angle annular dark field‐scanning TEM (HAADF‐STEM) pattern of post‐ORR NiSe_2_‐*V*_Se_ and its associated elemental EDX mapping images certificated the homogenous distribution of Ni and Se within this nanochain after ORR (Figure 5g–i and Figure S24, Supporting Information). EPR results for the cycled sample also showed the presence of Se vacancy with the same *g* value as the original one (Figure S25, Supporting Information). Based on the above findings, it could be rationally concluded that NiSe_2_‐*V*_Se_ catalyst possesses robust stability including composition, phase structure, morphology, and microstructure against the ORR cycling.

**Figure 6:**
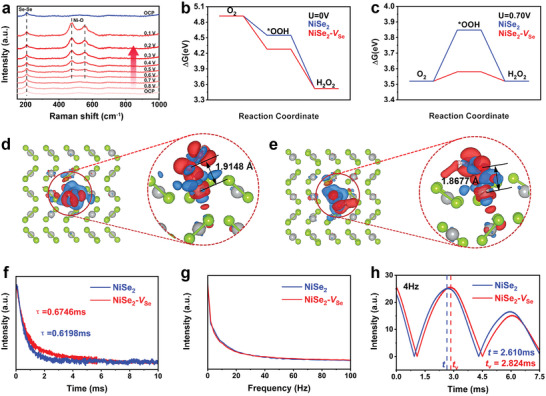
a) In situ Raman spectra (the red lines) for NiSe_2_‐*V*_Se_ electrocatalyst in O_2_‐saturated 0.1 m KOH within a voltage window 0.1–0.8 V versus RHE, together with the Raman spectrum (the blue line) back to OCP after forwarding direction test from 0.1–0.8 V versus RHE. Free energy diagram for 2e^−^ ORR on NiSe_2_ and NiSe_2_‐*V*_Se_ at b) *U* = 0 V and c) *U* = 0.70 V. Differential charge density distribution between adsorbed *OOH and d) NiSe_2_ and e) NiSe_2_‐*V*_Se_ substrates, where the grey and green sphere represent the Ni and Se atom, respectively, while the red and blue color isosurface means the positive and negative charge, respectively. f) TPV curves and the corresponding decay times of NiSe_2_ and NiSe_2_‐*V*_Se_, as well as their g) FFT patterns. h) Comparison of intensity‐time curves of NiSe_2_ and NiSe_2_‐*V*_Se_ (*t* and *t*_v_ represent the times when peak occurred for NiSe_2_ and NiSe_2_‐*V*_Se_, respectively).

To gain a deep understanding of the catalytic mechanism of NiSe_2_‐*V*_Se_ during the ORR process, in situ Raman measurements were carried out from 0.8 to 0.1 V (versus RHE) with an interval of 0.1 V. As shown in **Figure** **6**a, in the open circuit potential (OCP), only a band locating at around 206 cm^−1^ could be identified, which is associated to the Se—Se bond of selenides.^[^ ^50^ ^]^ Upon initiating the ORR, the Ni—O bands at around 478 and 553 cm^−1^ emerged due to the adsorption of —OOH on NiSe_2_‐*V*_Se_.^[^ ^51^, ^52^ ^]^ With the successive proceeding of ORR, these two peaks gradually augmented. These results suggested that the Ni sites in NiSe_2_‐*V*_Se_ served as the real active sites for the adsorption and desorption toward the key intermediate (*OOH) during 2e^−^ ORR. Interestingly, when the applied potential recovered back to open circuit potential (OCP), these two bands disappeared. Such a phenomenon also uncovered the high reversibility of active Ni atoms during ORR, thus contributing to the outstanding ORR activity, selectivity and stability.^[^ ^53^, ^54^ ^]^ Note that the band signal belonging to the Se—Se bond was preserved throughout the entire ORR process, further proving the phase stability of the NiSe_2_‐*V*_Se_ catalyst. Based on the in situ Raman results, DFT calculation was further performed to examine how Se vacancy enhances intrinsic 2e^−^ ORR activity and selectivity toward NiSe_2_ (Figure S26, Supporting Information). It is widely accepted that an ideal electrocatalyst that can satisfy the requirements of both excellent activity and selectivity should achieve an optimum balance between the adsorption and desorption of *OOH, that is, the *OOH adsorption strength should neither be too strong nor too weak, which can be directly reflected by the free adsorption energy of a catalyst toward *OOH (∆G_*OOH_, * means the catalytic site).^[^ ^48^, ^53^ ^]^ Therefore, the free energy diagrams during 2e^−^ ORR for both NiSe_2_ and NiSe_2_‐*V*_Se_ were plotted under the standard condition (*U* = 0 V, Figure 6b) and at the equilibrium potential of 2e^−^ ORR (*U* = 0.70 V, Figure 6c). Notably, compared with those of the pristine NiSe_2_ (4.548 and 3.848 eV at *U* = 0 and 0.70 V, respectively), NiSe_2_‐*V*_Se_ bear the closer ∆G_*OOH_ values (4.28 and 3.58 eV at *U* = 0 and 0.70 V, respectively) to the ideal ones (4.22 and 3.52 eV at *U* = 0 and 0.70 V, respectively).^[^ ^48^, ^54^ ^]^ Furthermore, the differential charge density distributions between the adsorbed *OOH and substrates (both NiSe_2_ and NiSe_2_‐*V*_Se_) were also simulated. By the comparison of Figures 6d and 6e, it could be seen that the negative‐charge polarized Se vacancy caused more apparent charge localization between adsorbed *OOH and catalytic slabs, by which the binding of active Ni site to *OOH was strengthened. Such a result was also in agreement with the upshift of the Ni *d*‐band center after introducing charge‐polarized Se vacancy, which was beneficial for improving the binding of active Ni site to intermediate, and thereby an optimization of ∆G_*OOH_. The energy diagram of NiSe_2_‐*V*_Se_ for the 4e^−^ ORR pathway from O_2_ to H_2_O was also calculated (Figure S27a, Supporting Information). Based on the previous reports,^[^ ^11^, ^55^ ^]^ the volcano curve based on the ∆G_*OH_ is depicted in Figure S27b, Supporting Information. According to the result, both NiSe_2_ and NiSe_2_‐*V*_Se_ preferred to undergo the 2e^−^ ORR pathway and the presence of Se vacancy is more beneficial for the whole performance of 2e^−^ ORR. In order to further demonstrate the advantage of the negative charge‐polarization, the Ni metal *d*‐band center as well as the free energy diagram during 2e^−^ ORR for NiSe_2_ with neutral charge Se vacancy (NiSe_2_‐*V*_se_‐N) is provided in Figure S28, Supporting Information. As expected, the Ni metal *d*‐band center for NiSe_2_‐*V*_se_‐N was −1.807 eV, which was located between −1.815 eV for NiSe_2_ and −1.790 eV for NiSe_2_‐*V*_Se_. This meant that the presence of Se vacancy could make an upshift of Ni metal *d*‐band, which would further shift to the Fermi level upon negative charge polarization in Se vacancy. Correspondingly, the ∆G_*OOH_ value for NiSe_2_‐*V*_se_‐N–OOH system was 3.61 eV under *U* = 0.70 V, which was much close to the ideal value of 3.52 eV than that of NiSe_2_–OOH system but was farther than that of NiSe_2_‐*V*_se_–OOH system, demonstrating again the positive role of negative charge polarization in Se vacancy for improving the intrinsic 2e^−^ ORR activity.

Apart from DFT calculations, the interfacial electron transfer process was also analyzed to gain insights into the electronic structure of NiSe_2_ tuned by Se vacancies on its ORR performance. Hence, we applied a stimulation‐response technique which was realized by a transient photo‐induced voltage (TPV), probing the electron transfer process at the interface. We plotted the TPV curves of NiSe_2_ and NiSe_2_‐*V*_Se_ in Figure 6f, both of which presented a similar attenuation trend, that is, as time passed, the TPV intensity decreased. However, the decay time of NiSe_2_‐*V*_Se_ was 0.6746 ms, higher than that of NiSe_2_ (0.6198 ms), implying that NiSe_2_‐*V*_Se_ had a slower overall electron transfer process which was believed to be triggered by Se vacancies. Generally, the sluggish electron transfer kinetics can promote the electrons to be remained at the surface of electrocatalysts for a longer duration instead of a quick and sufficient binding reaction with O_2_ molecules, thus blocking the 4e^−^ route for the subsequent ORR and elevating the H_2_O_2_ production performance.^[^ ^56^, ^57^ ^]^ This probably benefited from the Se vacancies present on NiSe_2_, which enabled the catalyst surface to trap electrons on the pristine NiSe_2_ surface. In order to deeply decouple the situations of electron transfer at the electrocatalyst surface, we further obtained the Fast Fourier Transform (FFT) curves derived from the TPV data, which displayed a series of continuous signals without any obvious peaks, illustrating that no distinct static and periodic frequency component appeared in the TPV relaxation signals (Figure 6g). On the other hand, the continuous wavelet transformation (CWT) curves were also performed for NiSe_2_ and NiSe_2_‐*V*_Se_ to explore various decay processes. As shown in Figure S29, Supporting Information, the as‐obtained 3D continuous wavelet transformation (CWT) patterns exhibited the relationship between the parameters of time, frequency and intensity, which could be used to analyze the TPV findings from the time‐scale/frequency‐scale. As observed, by comparing the relationship between peak intensity and time at varying frequencies, the dynamic electron transfer ability could be elaborated in detail, where the low and high frequency was defined as slow and fast electron transfer, respectively. Figure 6h shows respective peak positions at 2.610 (*t*) and 2.824 ms (*t*_v_) for NiSe_2_ and NiSe_2_‐*V*_Se_, respectively, under 4 Hz, demonstrating that NiSe_2_‐*V*_Se_ achieved the relatively slower transport of interfacial electrons at the low‐frequency region. Moreover, the inferior kinetics of interfacial electron transfer could also be found for NiSe_2_‐*V*_Se_ compared with NiSe_2_ under various frequencies which was progressively increased (Figure S30, Supporting Information). Also, at the fixed frequency, the time difference (Δ*t* = *t*_v_ − *t*) was also determined and compared to investigate the differences among various interfacial transport processes. At the frequency of 2, 4, 6, 8, 10, 12, 14 and 16 Hz, the associated Δ*t* values were 0.208, 0.214, 0.109, 0.077, 0.035, 0.030, 0.018 and 0 ms, respectively. Remarkably, the Δ*t* kept dropping to even zero with the increment of the frequency (Table S3, Supporting Information). Usually, the Δ*t* value decreases as the frequency increases, meaning that the most distinct difference in electron transport normally takes place in the low‐frequency region. Based on the above analysis, we could conclude that the presence of Se vacancies enabled NiSe_2_‐*V*_Se_ to possess a profoundly slower interfacial electron transport compared with NiSe_2_, which was beneficial to induce the surface electron trapping, thus modulating itself to follow a 2e^−^ ORR route.

## 3 Conclusions

In summary, nickel diselenide (NiSe_2_‐*V*_Se_) with charge‐polarized anion vacancy was synthesized via a sequential solvothermal–calcination–selenization–annealing process and was demonstrated as an efficient electrocatalyst for electrosynthesis of H_2_O_2_ through 2e^−^ ORR. The as‐prepared NiSe_2_‐*V*_Se_ presented an outstanding 2e^−^ ORR catalytic activity, which delivered a maximum disk current density (2.94 mA cm^−2^) at a high potential of 0.3 V (versus RHE) and a remarkable H_2_O_2_ selectivity as high as 96% at 0.45 V (versus RHE). Most importantly, the exceptional catalytic activity and selectivity decreased only slightly even in 40 000s and 5000 ADT cycles. The impressive performance achieved for NiSe_2_‐*V*_Se_ was much better than that of pristine NiSe_2_, and also one of the best among the previously reported Ni‐based electrocatalysts for 2e^−^ ORR in alkaline media. Further in situ and ex situ investigations combined with theoretical calculations unveiled that Se vacancies with negative charges not only shift the *d*‐band center in the upward direction accompanied by the release of lattice strain but also induced a distinct surface electron trap effect. Such a vacancy inducement improves structural stability, suppressing the competitive 4e^−^ ORR pathway as well as optimizing the intrinsic adsorption free energy toward *OOH intermediate for the selective production of H_2_O_2_. Therefore, this contribution explicitly demonstrates the integration of selenide with charge‐polarized anion vacancy as well as the related findings for the application of ORR to produce H_2_O_2_ and establishes NiSe_2_‐*V*_Se_ as the new benchmark 2e^−^ ORR electrocatalysts in alkaline solution. Finally, this study provides clear fundamental insights and offers a new design platform for cost‐effective, stable, and efficient earth‐abundant transition metal‐based electrocatalysts.

## Conflict of Interest

The authors declare no conflict of interest.

## Supporting information

Supporting Information

## Data Availability

The data that support the findings of this study are available in the supplementary material of this article.
