# Supplementary material for: Charge‐Polarized Selenium Vacancy in Nickel Diselenide Enabling Efficient and Stable Electrocatalytic Conversion of Oxygen to Hydrogen Peroxide
Source: Adv Sci (Weinh). 2022 Dec 7;10(4):2205347. doi: 10.1002/advs.202205347 (PMC9896043; doi:10.1002/advs.202205347)
Supplement: Supplementary file 1 — Supporting Information [file ADVS-10-2205347-s001.pdf]

# Supporting Information

**Charge-Polarized Selenium Vacancy in Nickel Diselenide Enabling Efficient and Stable Electrocatalytic Conversion of Oxygen to Hydrogen Peroxide**

*Yingming Wang, Hui Huang, Jie Wu, Hongyuan Yang, Zhenhui Kang,* Yang Liu, Zhaowu Wang, Prashanth W. Menezes,* Ziliang Chen**

Mr. Y. Wang, Mr. J. Wu, Dr. H. Huang, Prof. Y. Liu, Prof. Z. Kang, Dr. Z. Chen
Institute of Functional Nano & Soft Materials (FUNSOM), Jiangsu Key Laboratory for Carbon-Based Functional Materials & Devices, Soochow University, 199 Ren'ai Road, Suzhou, 215123, Jiangsu, China.
E-mail: zhkang@suda.edu.cn, zlchen@suda.edu.cn

Mr. H. Yang, Dr. P. W. Menezes
Department of Chemistry: Metalorganics and Inorganic Materials, Technische Universität Berlin, Straße des 17 Juni 135, Sekr. C2, 10623 Berlin, Germany
E-mail: prashanth.menezes@mailbox.tu-berlin.de

Dr. P. W. Menezes
Material Chemistry Group for Thin Film Catalysis – CatLab, Helmholtz-Zentrum Berlin für Materialien und Energie, Albert-Einstein-Str. 15, 12489 Berlin, Germany
E-mail: prashanth.menezes@helmholtz-berlin.de

Dr. Z. Wang
School of Physics and Engineering, Henan University of Science and Technology, Luoyang, 471023, China

**Experimental section**

**Chemicals**

Nickel nitrate hexahydrate ($Ni(NO_3)_2 \cdot 6H_2O$) was purchased from Aladdin Ltd (Shanghai, China). Hexamethylene tetramine (HMT) was purchased from Alfa Aesar. Selenium (Se) was purchased from Aladdin Ltd (Shanghai, China). Nafion PFSA Polymer Dispersions D520 (5%) was purchased from Sinero. The water used throughout all experiments was purified through a Millipore system. All the chemicals were used as received without further purification.

**Preparation of $NiSe_2$ nanoparticles**

$NiSe_2$ was prepared as follows. In a typical procedure, 5 mmol $Ni(NO_3)_2 \cdot 6H_2O$ and 10 mmol HMT were dissolved in 40 mL distilled water and stirred to form a clear solution. Then the aqueous solution was transferred to a 50 ml Teflon-lined stainless-steel autoclave. It was heated at 100 ºC for 10 h to achieve $Ni(OH)_2$. After the autoclave cooled down naturally, the resulting precipitate was taken out and washed with distilled water and ethanol several times alternatively, followed by drying for 6 h at 60 ºC to obtain the hydroxide precursor. The precursor was then placed in a muffle furnace and heated at 500 °C for 3h at a heating rate of 10 ℃ $min^{-1}$ to obtain the NiO. Next, Se (1 g) was placed in the tube at the farthest upstream position and the NiO precursor was placed at the center of the furnace. The sample was heated at 500 ºC for 3 h with a heating speed of 10 ºC $min^{-1}$ under $N_2$ atmosphere and then cooled to room temperature naturally. Finally, the black $NiSe_2$ nano-particles were collected for further characterization.

**Preparation of $NiSe_2$-$V_{Se}$ nanoparticles**

The $NiSe_2$ black powders prepared by the above method were put into a tubular furnace, heated at a rate of 5 ºC $min^{-1}$ under $N_2$ atmosphere, and kept at 350 ºC for 30 min to obtain $NiSe_2$-$V_{Se}$. Other control samples were annealed under the same condition except the annealing temperature was 250 ºC, 300 ºC, 400 ºC, and 450 ºC, respectively.

**Characterization**

The phase structure and abundance of as-prepared samples were obtained by powder X-ray diffraction (XRD) measurements on a D8 ADVANCE X-ray diffractometer with Cu Kα radiation ($\lambda$ = 1.5406 Å). The XRD profiles were further analyzed by the Rietveld refinement program RIETAN-FP and the lattice strain based on the Halder-Wagner method was also

extracted by RIETAN-FP stimulation.[1] The element chemical states of samples were characterized by X-ray photoelectron spectrometry (XPS), which was carried out on an ESCALAB 250Xi spectrometer (Thermo Scientific, USA) equipped with a pass energy of 30 eV with a power of 100 W (10 kV and 10 mA) and a mono-chromatized AlKα X-ray (hν=1486.65 eV) source. All samples were analyzed under a pressure of less than $1.0\times10^{-9}$ Pa. Spectra were acquired through the advantage software (Version 5.979) with a step of 0.05 eV. The morphology and microstructure of samples were investigated by field-emission scanning electron microscope (FESEM, Zeiss G500) and transmission electron microscope (TEM, FEI Talos F200X). *In-situ* Raman spectra during the ORR process were recorded by a Raman spectrometer with an excitation wavelength of 532 nm (Horiba LabRAM HR Evolution).

**Electrochemical measurements**

Firstly, 3.6 mg as-prepared electrocatalyst and 0.4 mg Ketjen Black were mixed and dispersed in a mixture solution containing 600 μL $H_2O$, 300 μL ethanol and 100 μL 0.5 wt.% Nafion solution. In order to obtain a uniform ink, the mixed solution was sonicated in an ice water bath for 30 min. Then 6.20 μL of the ultrasonic solution was dripped on the glassy carbon of the rotating ring disk electrode (RRDE) with an area of 0.1256 $cm^2$. The RRDE (RRDE-3A, 012613 Fixed-Disk, Japan) consists of a glassy carbon rotation disk (disk OD = 4.0 mm) and a Pt ring (ring OD = 7.0 mm). The theoretical collection efficiency of RRDE is 37%. An electrochemical workstation (760E, CHI) was used to evaluate the electrochemical performance of the catalysts. A standard three-electrode system was used to evaluate the performance of the catalyst, where the RRDE loaded with catalysts, Hg/HgO and graphite rod served as the working electrode, reference electrode and counter electrode, respectively. The electron transfer number ($n$) and $H_2O_2$ selectivity ($H_2O_2$%) were calculated as follows:

$$n = 4\frac{I_D}{I_D + I_R/N} \tag{1}$$

$$H_2O_2(\%) = 200\frac{I_R / N}{I_D + I_R / N} \tag{2}$$

where $I_R$ represented the ring current, $I_D$ represented the absolute value of disk current and the collection efficiency ($N$) of the RRDE was determined as 0.37.

**Electrochemical measurements in H-cell electrolyzer.**

The $H_2O_2$ yield of the catalyst in 0.1 M KOH was first determined using an H-cell electrolyzer, in which the catalyst-loaded carbon paper was used as the working electrode (0.5 mg $cm^{-2}$), and the Pt foil was used as the counter electrode. Catalyst ink was prepared by mixing 5 mg catalyst, 100 μL 5 wt % Nafion with 600 μL $H_2O$ and 300 μL ethanol. All the electrochemical experiments were tested at a CHI760 (Chenhua, Shanghai) electrochemical workstation. The reactions were tested by chronopotentiometry at 0.4 V vs. RHE without ohmic drop correction. The $H_2O_2$ productivity was determined by potassium permanganate ($KMnO_4$) titration. Specifically, 2 mL extracting catholyte was firstly acidified by 2 mL $H_2SO_4$, followed by titrating using 0.02 mol $L^{-1}$ $KMnO_4$. Note when the last drop of $KMnO_4$ standard solution was added, the color change of the acidified catholyte initiated and remained for half a minute when the total amount of $H_2O_2$ was calculated by the consumption of $KMnO_4$. The Faraday efficiency (FE) and yield rate (YR) for $H_2O_2$ generation in H-cell was calculated as follows:

$$FE(\%) = \frac{2 \times 96500 \times M_{H_2O_2}}{C} \times 100\% \tag{3}$$

$$YR = \frac{M_{H_2O_2}}{A \times t \times g_{cat}} \tag{4}$$

Where $M_{H_2O_2}$ is the mole of generated $H_2O_2$, $C$ is the totally consumed charges, $A$ is the area of the cathode electrode, t is the electrolysis duration and $g_{cat}$ is the mass of the catalyst.

**TPV principle**

A stimulation-response method, transient phototelepressure (TPV) measurement, was performed under room temperature using a homemade measurement system, in which a platinum mesh was covered with powder samples (1 * 1cm) and a platinum wire served as the working and counter electrode, respectively. The powder samples were irradiated by a laser generated by a third harmonic Nd:YAG laser (Polaris II, New Wave Research, Inc.) radiation pulse (λ = 355 nm, pulse width 5 ns). Accordingly, the generated photocurrent was first recognized and amplified. Hereafter, the oscilloscope recorded the photocurrent based on the ratio of the photovoltage to the internal resistance of the test system.

**Theoretic calculations**

The density functional theory calculations were carried out by Vienna ab-Initio Simulation Package.[1] Core electrons are described by pseudopotentials generated from the projector

augmented wave method,[2] and valence electrons are expanded in a plane-wave basis set with an energy cutoff of 450 eV. The Perdew-Burke- Ernzerh (PBE) exchange correlation functional was used. Based on the TEM observation, (200) surface was selected for $NiSe_2$ and was modeled by a (2×2) supercell consisting of 96 atoms and a vacuum of 18 Å. The bottom six atom layers were fixed to mimic the bulk and the other six layers were fully relaxed. For the sampling of Brillouin-zone integrals, Gamma centered k-points grid of 3×3×1 was used for the slab model. During structure optimation, all energy change criterion was set to $10^{-4}$ eV, the atoms were relaxed until the force acting on each atom was less than 0.02 eV $Å^{-1}$. One Se atom on the surface was removed to introduce Se vacancy. It is found that the Se vacancy of the outermost layer has the lowest energy which was used to study the ORR property. The ORR process was studied by calculating the free energy diagram of $2e^-$ and $4e^-$ ORR pathways proposed by Norskov and coworkers.[3-5] The reaction free energy $\Delta G$ for each step was defined as the difference between free energies of the initial and final states and was calculated by the following equation:

$$\Delta G = \Delta E + \Delta ZPE - T\Delta S + \Delta G_U + \Delta G_{pH} \quad (5)$$

Where $\Delta E$ represented the reaction energy of reactant molecules adsorbed on the catalyst surface; *ZPE* and *S* meant the zero-point energy and entropy, respectively; $\Delta G_U = -neU$; $\Delta G_{PH}$ was the correction of the $H^+$ free energy.

**Figures**

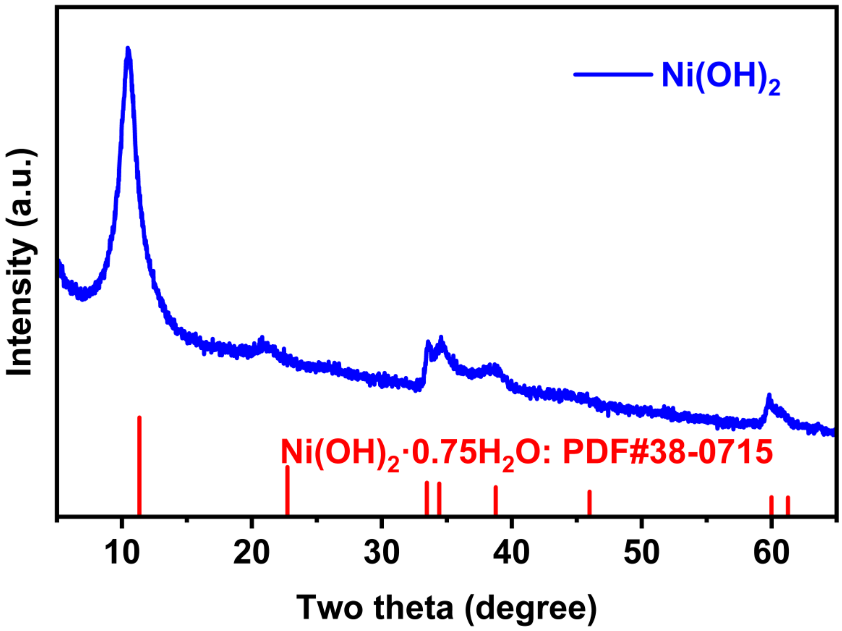


**Figure S1**. The XRD pattern of $Ni(OH)_2$ compound.

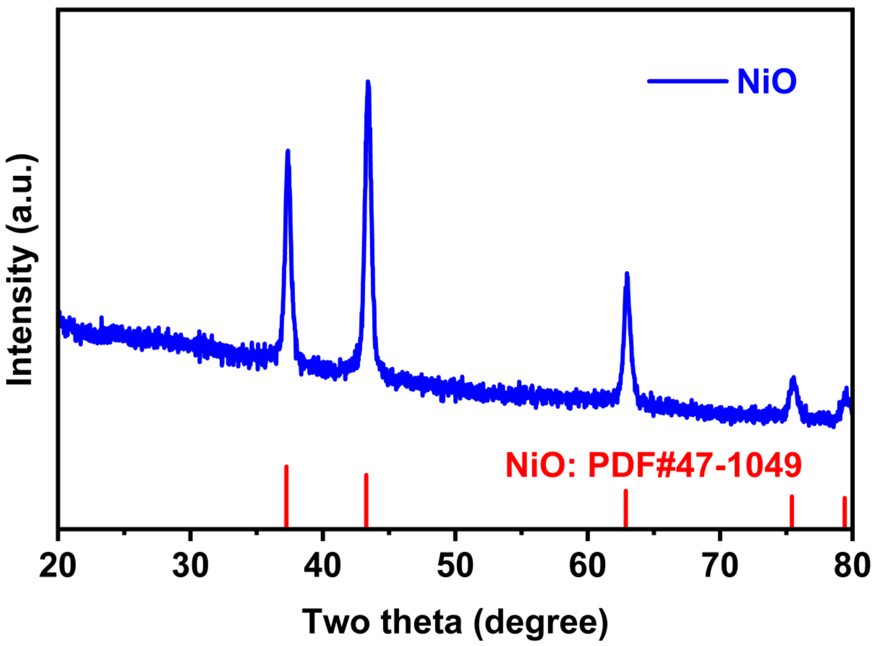


**Figure S2**. The XRD pattern of NiO compound.

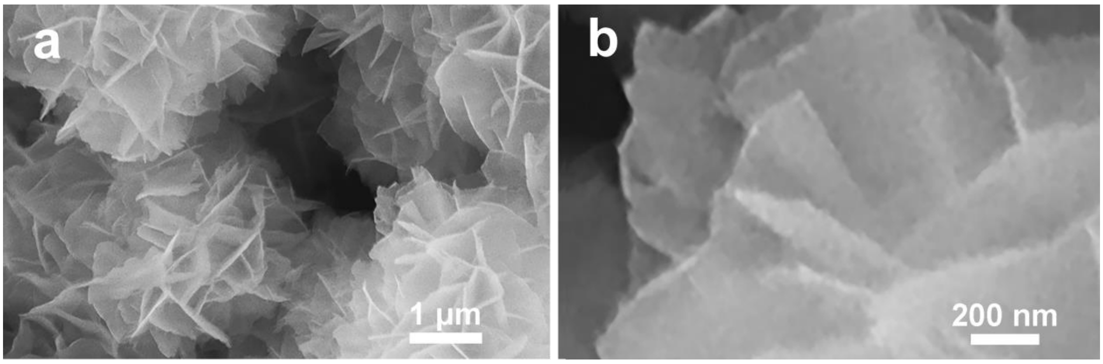


**Figure S3**. (a, b) The FESEM images of $Ni(OH)_2$ nanoflower.

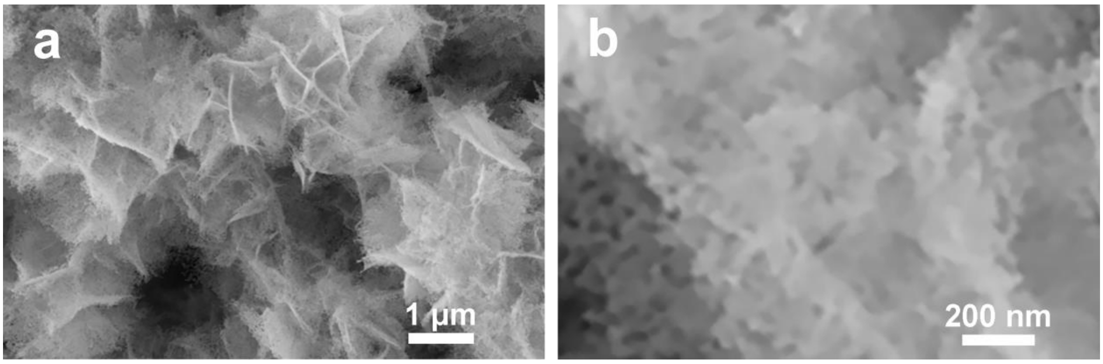


**Figure S4**. (a, b) The FESEM images of NiO nanoflower.

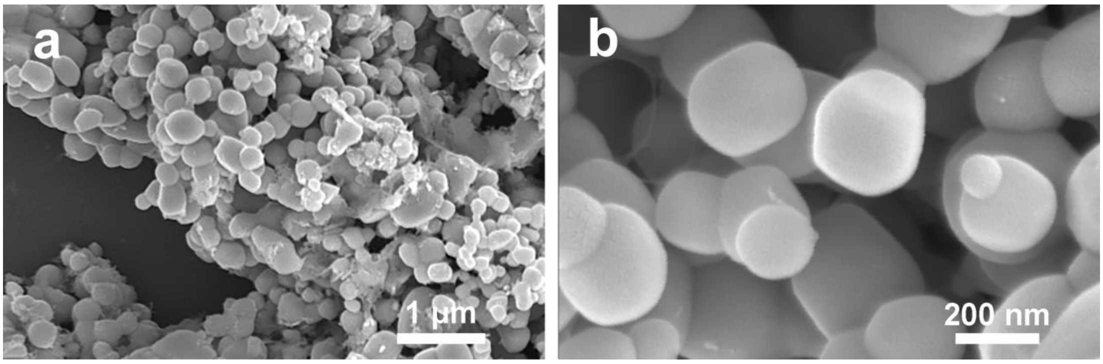


**Figure S5**. (a, b) FESEM images of pristine $NiSe_2$ nanochain.

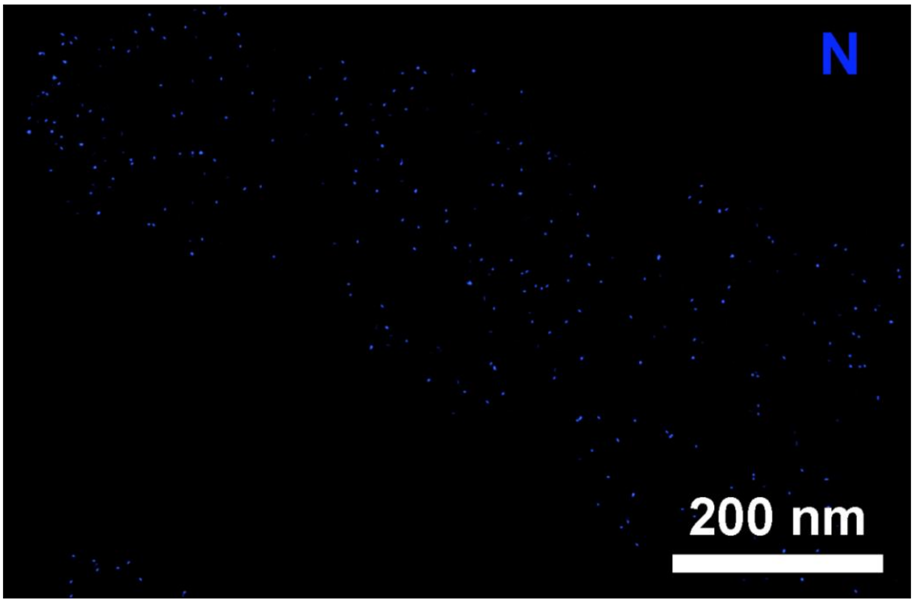


**Figure S6**. $NiSe_2$-$V_{Se}$ particle EDX elemental mapping of N.

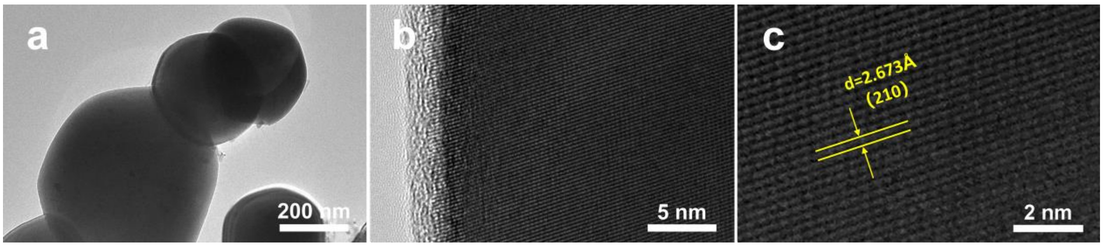


**Figure S7**. (a) The TEM, (b) high-magnified, and (c) high-resolution (HR) TEM images of $NiSe_2$.

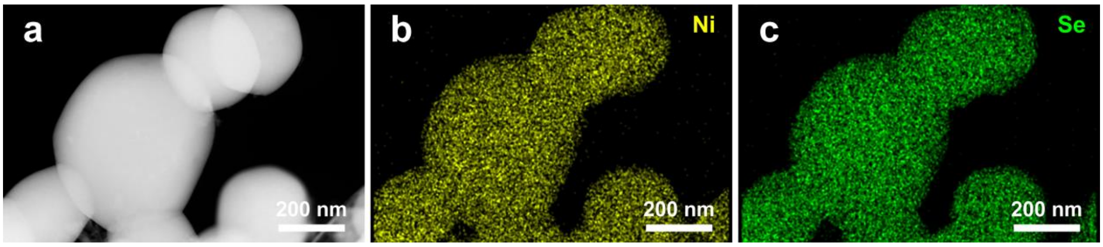


**Figure S8**. (a) HAADF pattern of representative $NiSe_2$ particles and the corresponding EDX elemental mapping of (b) Ni, and (c) Se species.

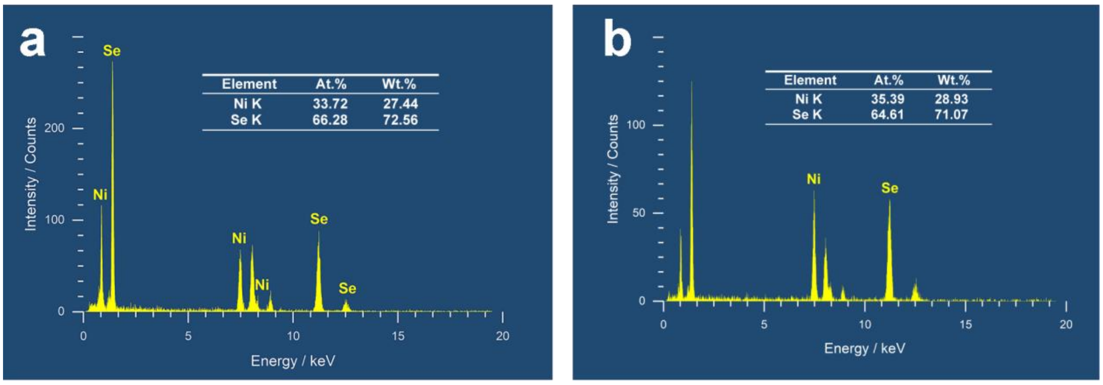


**Figure S9**. TEM-EDX mapping spectra of (a) pristine $NiSe_2$ and (b) $NiSe_2$-$V_{Se}$ with the corresponding mass fraction and atomic ratio.

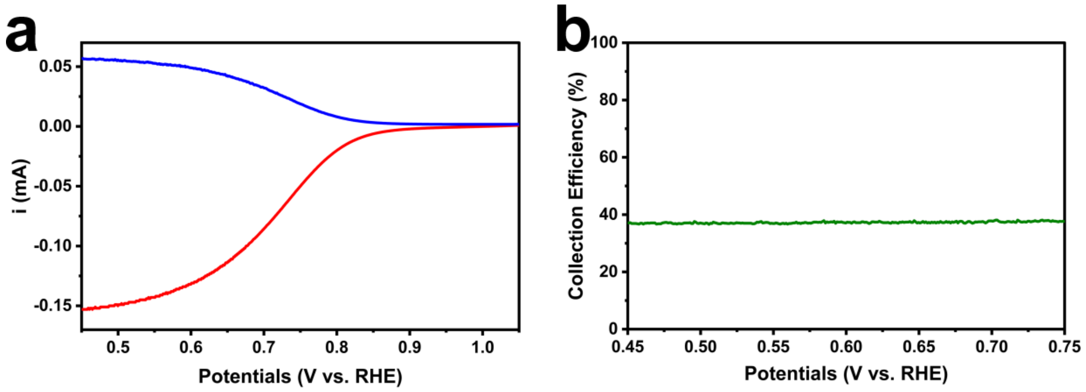


**Figure S10.** Calibration of the collection efficiency of the bare ring disk electrode (RRDE) in Ar-saturated 1 M $KNO_3$ dissolved with 2 mM $K_3[Fe(CN)_6]$. (a) RRDE voltammograms recorded by performing LSV on disk from 0.45 V to 1.05 V at 10 mV $s^{-1}$ at 1600 rpm while holding the ring at 0.64 V vs. RHE. (b) The corresponding collection efficiency of RRDE voltammograms as a function of the potential.

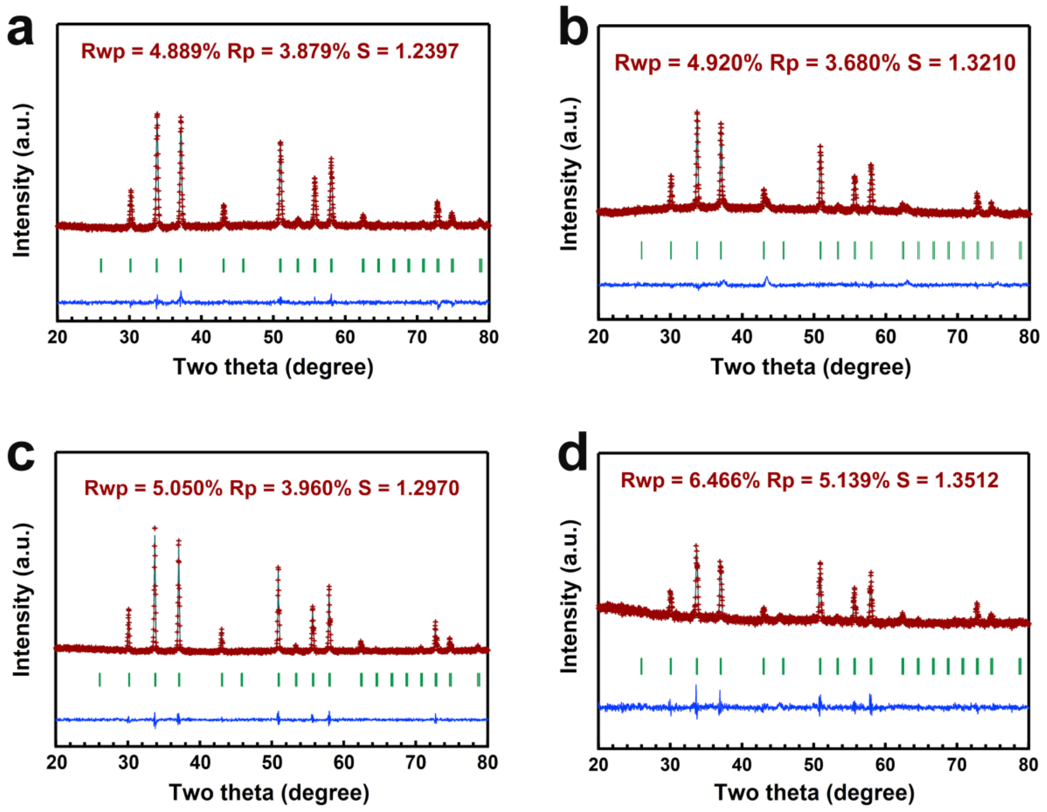


**Figure S11.** The Rietveld refinement of XRD patterns for (a) $NiSe_2$-$V_{Se}$-250, (b) $NiSe_2$-$V_{Se}$-300 $NiSe_2$-$V_{Se}$-300, (c) $NiSe_2$-$V_{Se}$-400 and (b) $NiSe_2$-$V_{Se}$-450.

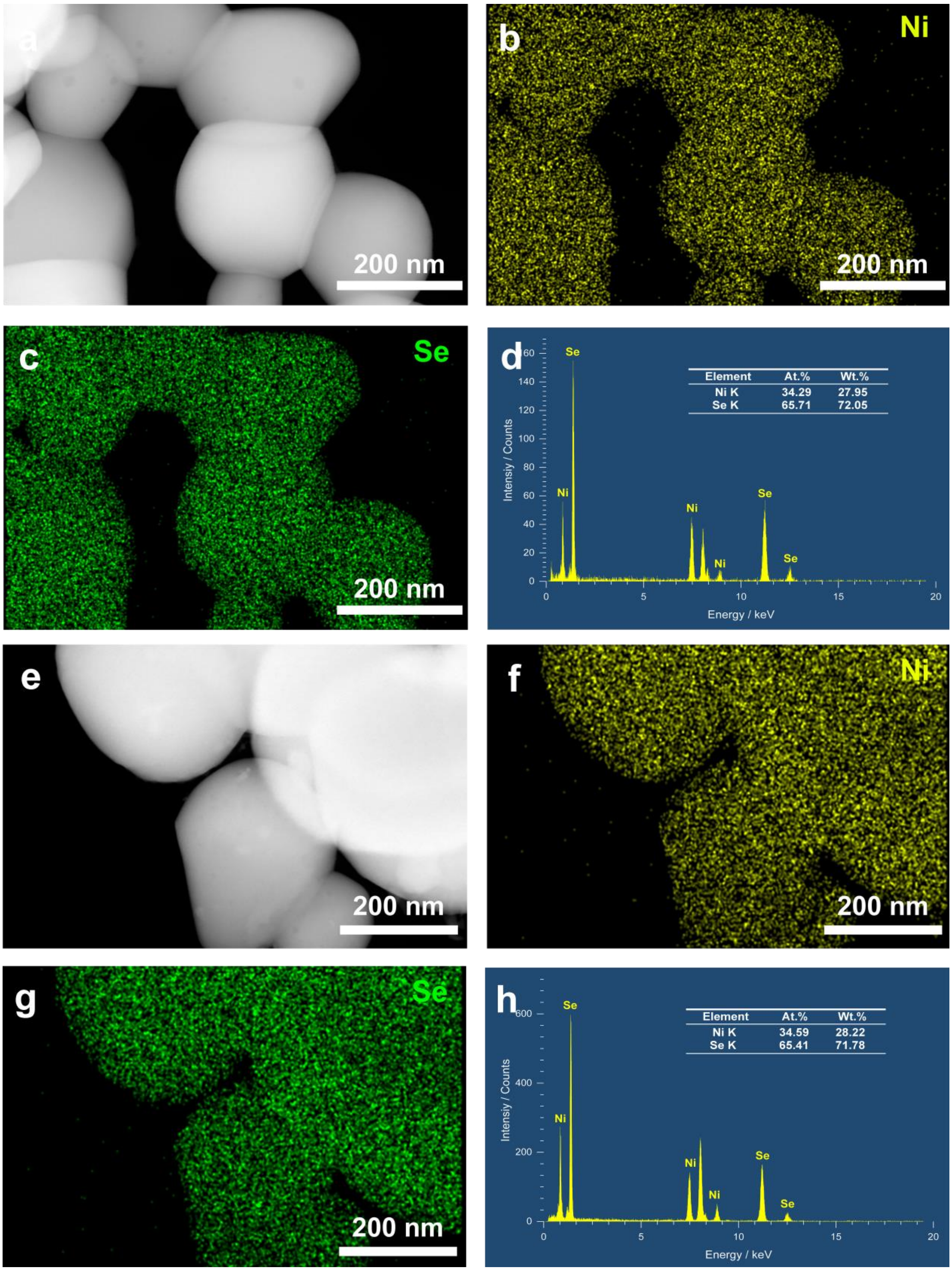


**Figure S12.** (a) HAADF pattern of representative $NiSe_2$-$V_{Se}$-250 particles and the corresponding elemental mapping of (b) Ni, and (c) Se species as well as (d) EDX spectra; (e) HAADF pattern of representative $NiSe_2$-$V_{Se}$-300 particles and the corresponding elemental mapping of (f) Ni, and (g) Se species as well as (h) EDX spectra.

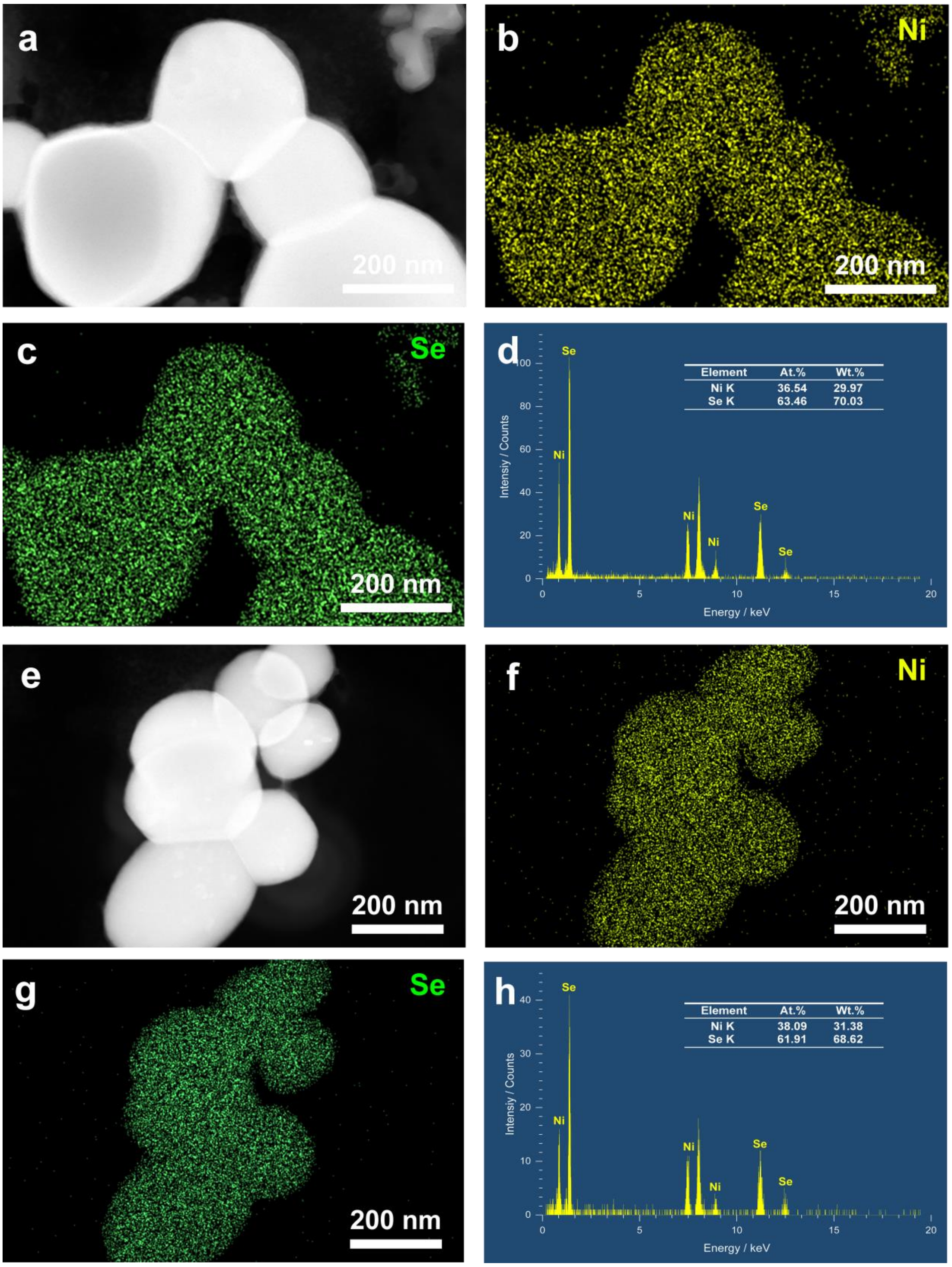


**Figure S13.** (a) HAADF pattern of representative $NiSe_2$-$V_{Se}$-400 particles and the corresponding elemental mapping of (b) Ni, and (c) Se species as well as (d) EDX spectra; (e) HAADF pattern of representative $NiSe_2$-$V_{Se}$-450 particles and the corresponding elemental mapping of (f) Ni, and (g) Se species as well as (h) EDX spectra.

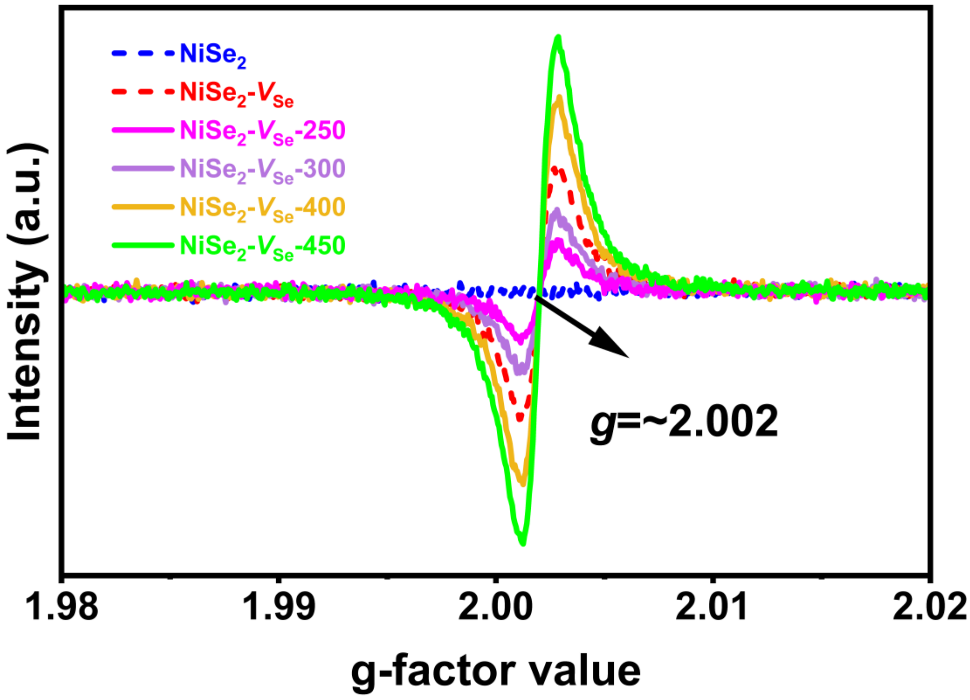


**Figure S14.** EPR spectra of $NiSe_2$, $NiSe_2$-$V_{Se}$, $NiSe_2$-$V_{Se}$-250, $NiSe_2$-$V_{Se}$-300, $NiSe_2$-$V_{Se}$-400 and $NiSe_2$-$V_{Se}$-450.

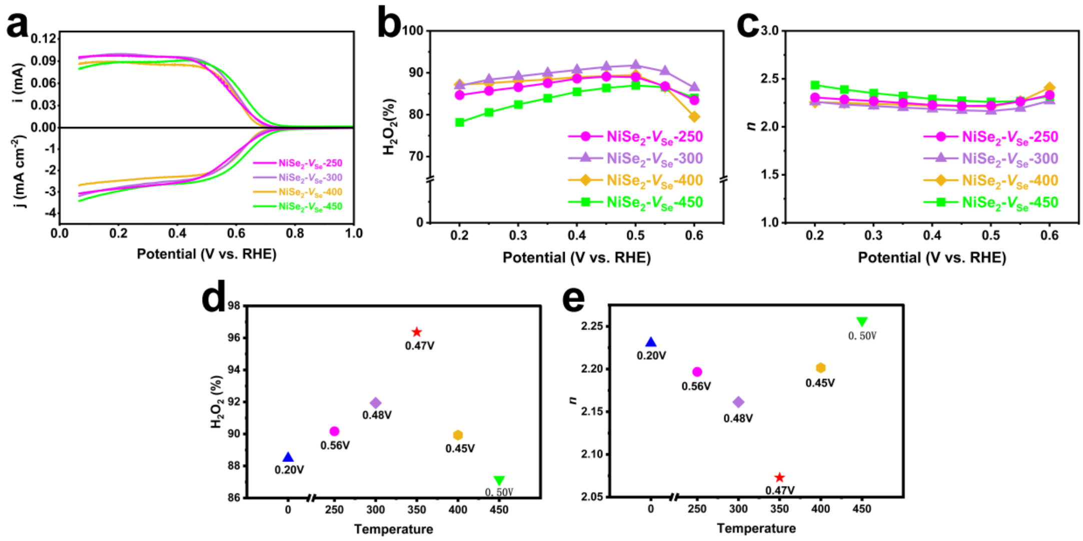


**Figure S15.** (a) LSV curves of $NiSe_2$-$V_{Se}$-250, $NiSe_2$-$V_{Se}$-300, $NiSe_2$-$V_{Se}$-400 and $NiSe_2$-$V_{Se}$-450 recorded at 1600 rpm with a scan rate of 10 mV $s^{-1}$ (bottom part), together with the corresponding $H_2O_2$ current on the ring electrode (upper part). (b) Selectivity of $H_2O_2$ and (c) calculated electron transfer number ($n$) within the potential sweep. The comparison of (d) the highest selectivity of $H_2O_2$ and (e) the lowest number of transferred electrons at different annealing temperatures

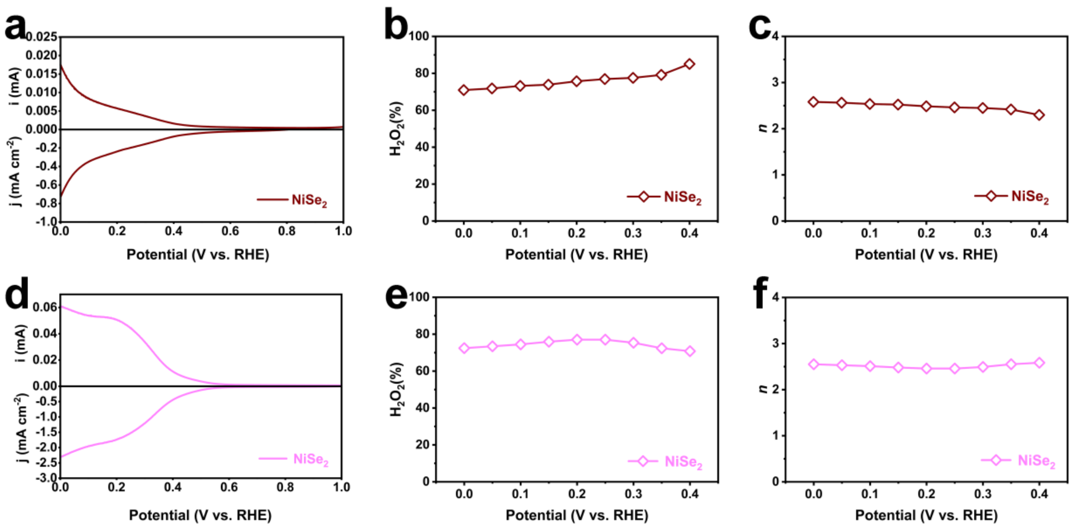


**Figure S16.** (a) LSV curves of the $NiSe_2$ sample in 0.05 M $H_2SO_4$, where the disk current density ($j_{disk}$) together with the ring currents ($i_{ring}$) was fixed at a potential of 1.50 V *vs.* RHE; (b) Selectivity of $H_2O_2$ (%) and (c) electron transfer number (*n*) calculated from the corresponding LSV curve; (d) LSV curves of the $NiSe_2$ sample in 0.1 M $Na_2SO_4$, where the disk current density ($j_{disk}$) together with the ring currents ($i_{ring}$) was fixed at a potential of 1.50 V *vs.* RHE; (e) Selectivity of $H_2O_2$ (%) and (f) electron transfer number (*n*) calculated from the corresponding LSV curve.

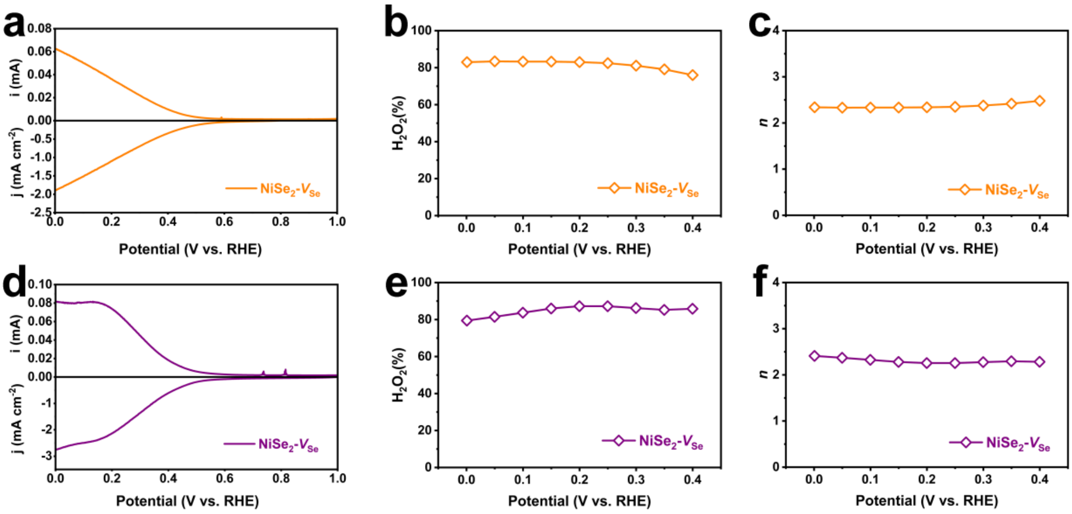


**Figure S17.** (a) LSV curves of the NiSe$_2$-$V_{Se}$ sample in 0.05 M $H_2SO_4$, where the disk current density ($j_{disk}$) together with the ring currents ($i_{ring}$) was fixed at a potential of 1.50 V *vs.* RHE; (b) Selectivity of $H_2O_2$ (%) and (c) electron transfer number (*n*) calculated from the corresponding LSV curve; (d) LSV curves of the NiSe$_2$-$V_{Se}$ sample in 0.1 M $Na_2SO_4$, where the disk current density ($j_{disk}$) together with the ring currents ($i_{ring}$) was fixed at a potential of 1.50 V *vs.* RHE; (e) Selectivity of $H_2O_2$ (%) and (f) electron transfer number (*n*) calculated from the corresponding LSV curve.

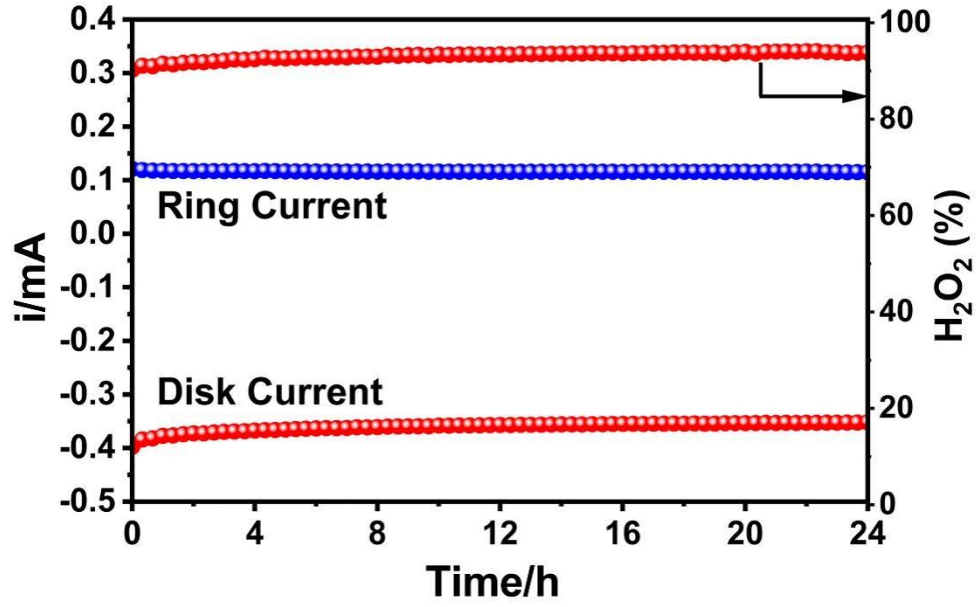


**Figure S18.** Stability tests of $NiSe_2$-$V_{Se}$ at a fixed disk potential of 0.45 V (*vs*. RHE).

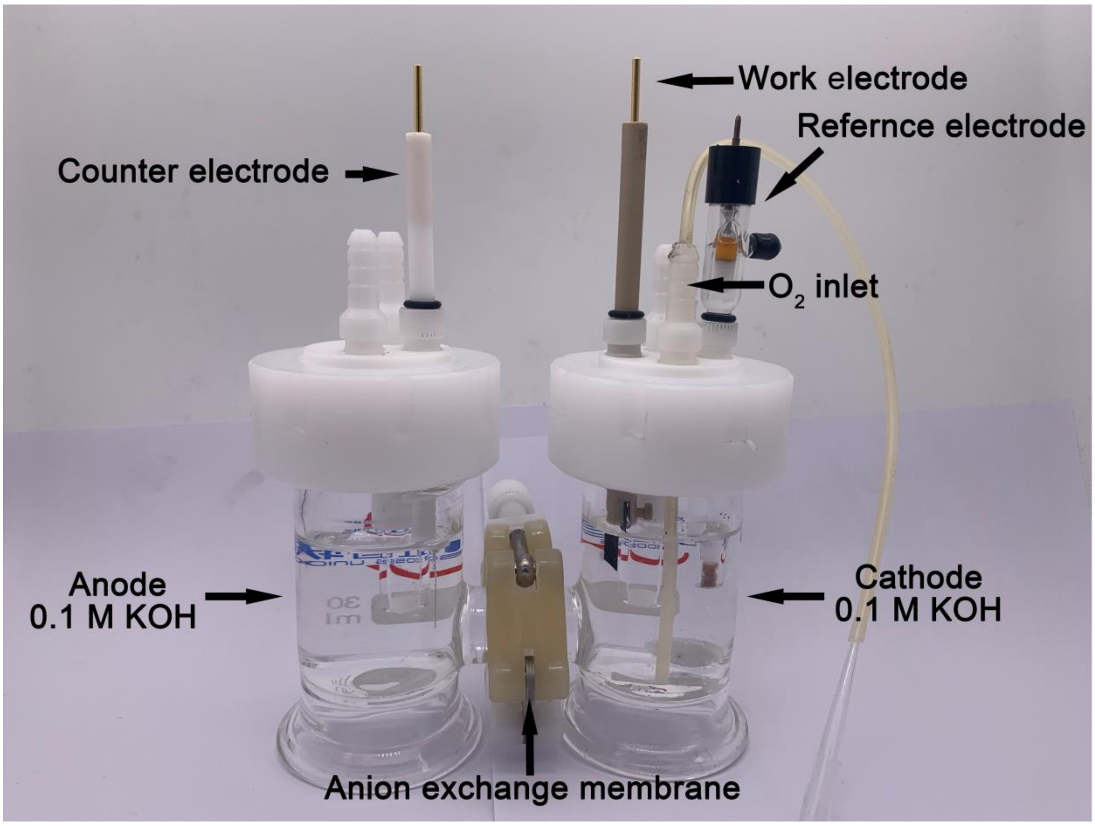


**Figure S19**. Optical image of H-type electrolytic cell.

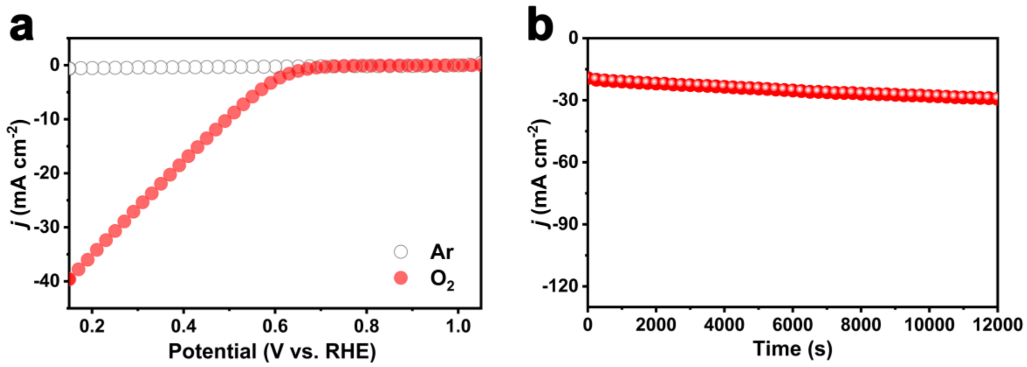


**Figure S20**. (a) LSV curve of 0.5 mg cm$^{-2}$ $NiSe_2$-$V_{Se}$ catalyst supported on 1*1 cm$^2$ carbon paper, and (b) the corresponding chronoamperometry curve at 0.4 V *vs.* RHE in an $O_2$-saturated electrolyte for 12000 s.

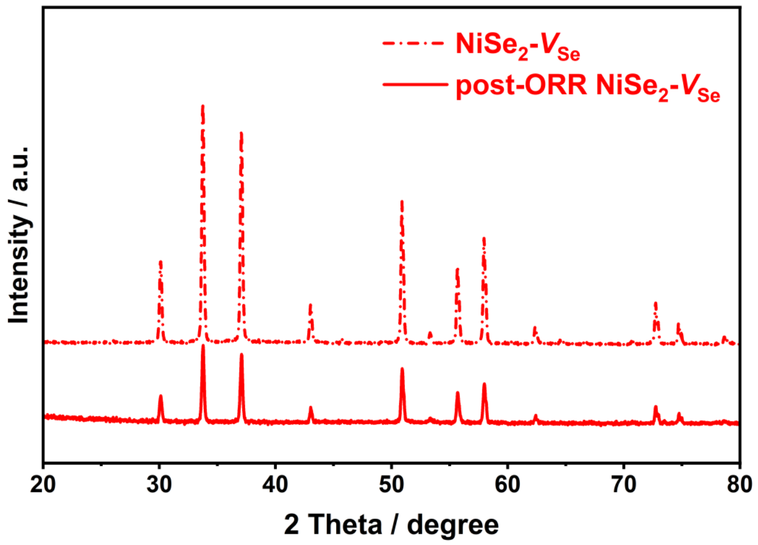


**Figure S21**. The XRD patterns of $NiSe_2$-$V_{Se}$ and post-ORR $NiSe_2$-$V_{Se}$.

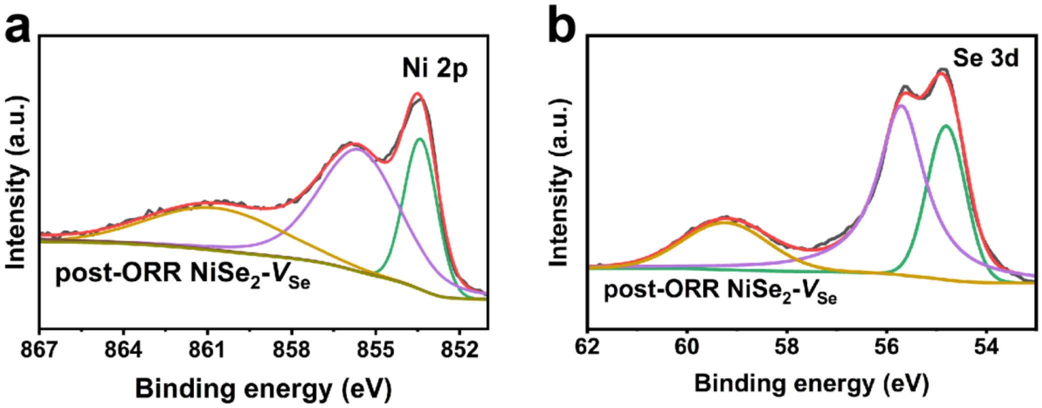


**Figure S22**. The high-resolution XPS spectra of (a) Ni 2p and (b) Se 3d in post-ORR $NiSe_2$-$V_{Se}$ compound.

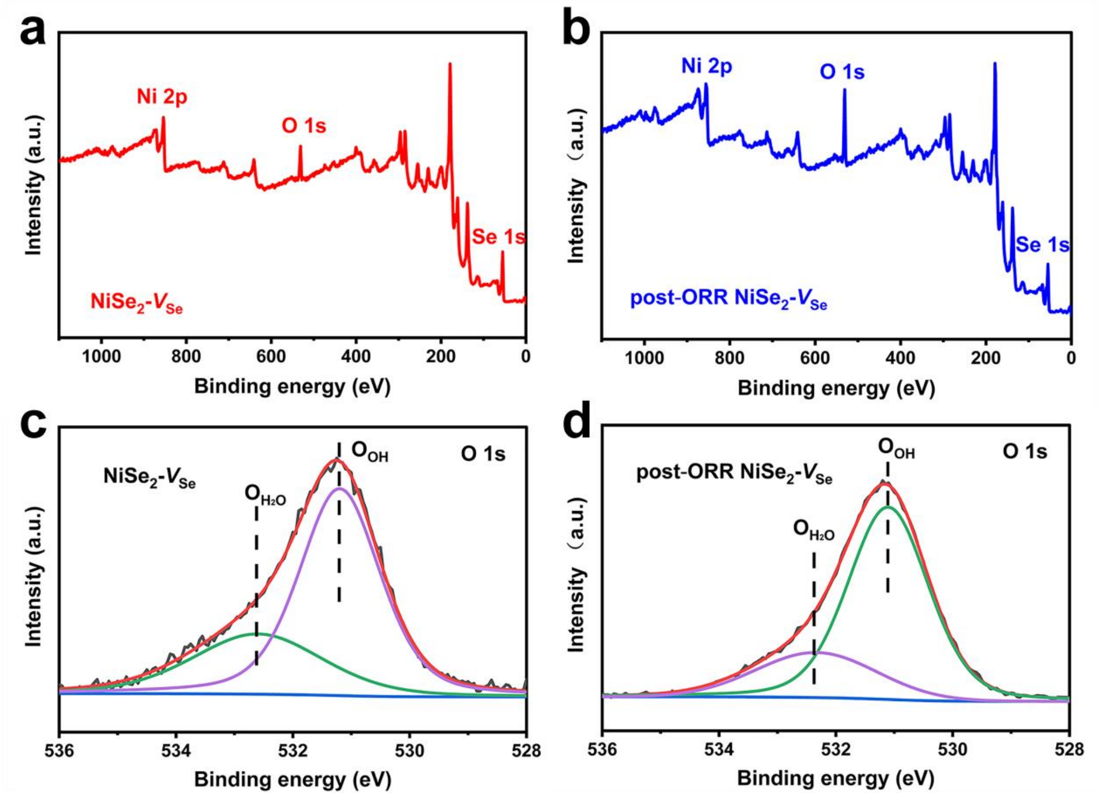


**Figure S23**. XPS survey spectra of (a) $NiSe_2$-$V_{Se}$ and (b) post-ORR $NiSe_2$-$V_{Se;}$ High-resolution XPS spectra of O 1s in (c) $NiSe_2$-$V_{Se}$ and (d) post-ORR $NiSe_2$-$V_{Se}$.

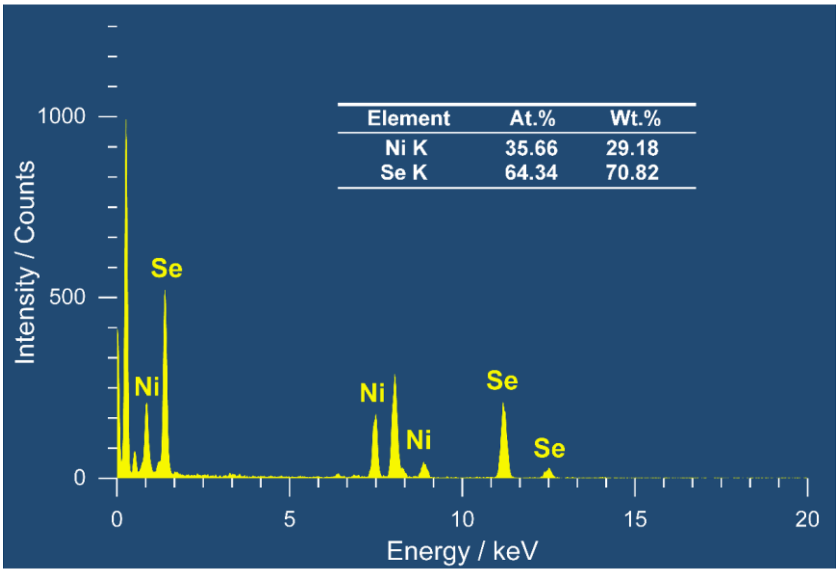


**Figure S24**. TEM-EDX result of post-ORR $NiSe_2$-$V_{Se}$ with the corresponding mass fraction and atomic ratio.

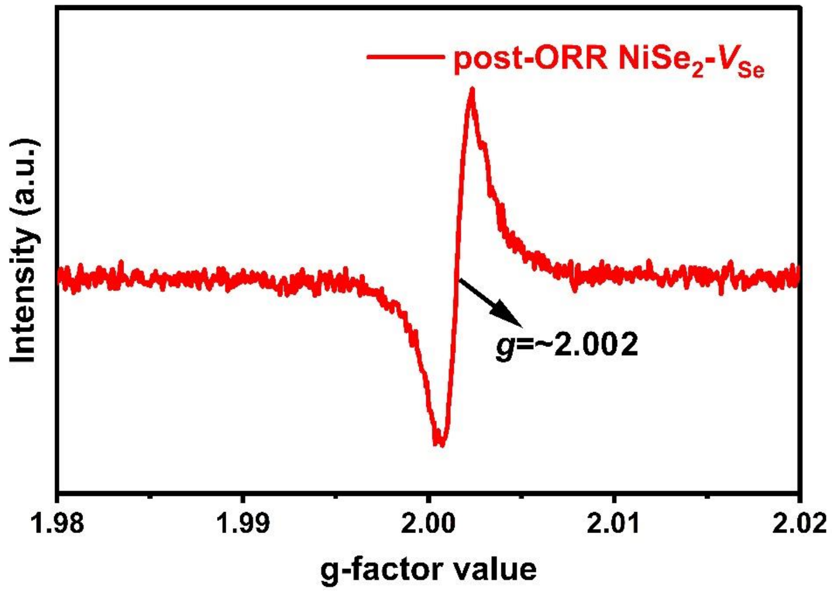


**Figure S25**. EPR spectrum of post-ORR $NiSe_2$-$V_{Se}$.

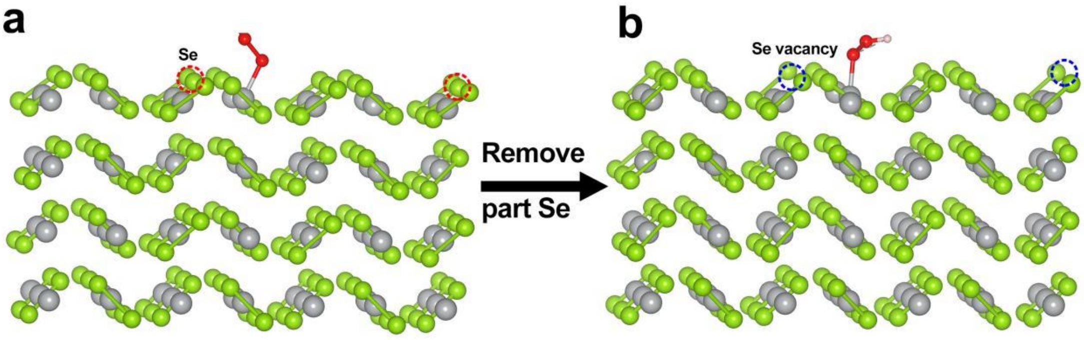


**Figure S26**. The DFT calculation models of (a) $NiSe_2$–OOH and (b) $NiSe_2$-$V_{Se}$–OOH, where the grey, green, red, and pink spheres represent the Ni, Se, O, and H atom, respectively.

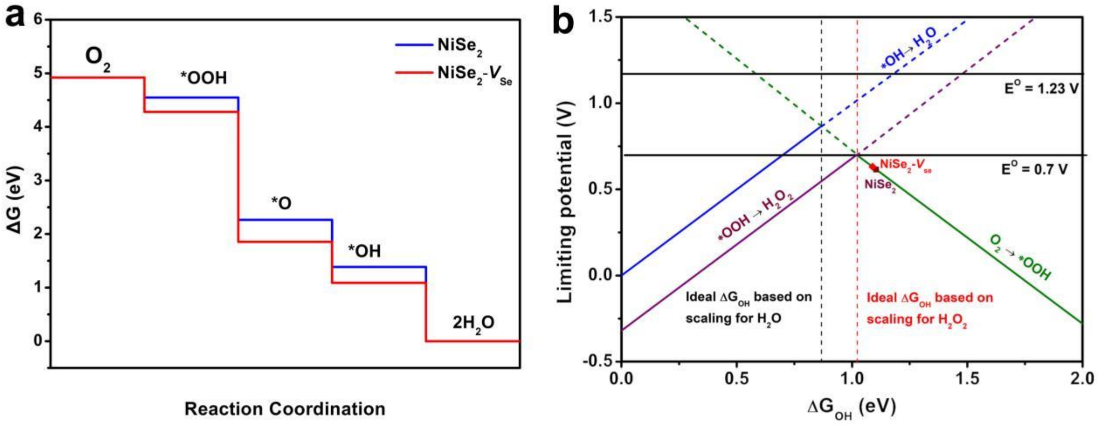


**Figure S27**. (a) Energy diagram for $NiSe_2$ and $NiSe_2$-$V_{Se}$ towards $4e^-$ ORR; (b)Volcano plot for ORR process based on $\Delta G_{OH}$, where $NiSe_2$ and $NiSe_2$-$V_{Se}$ for ORR are shown.

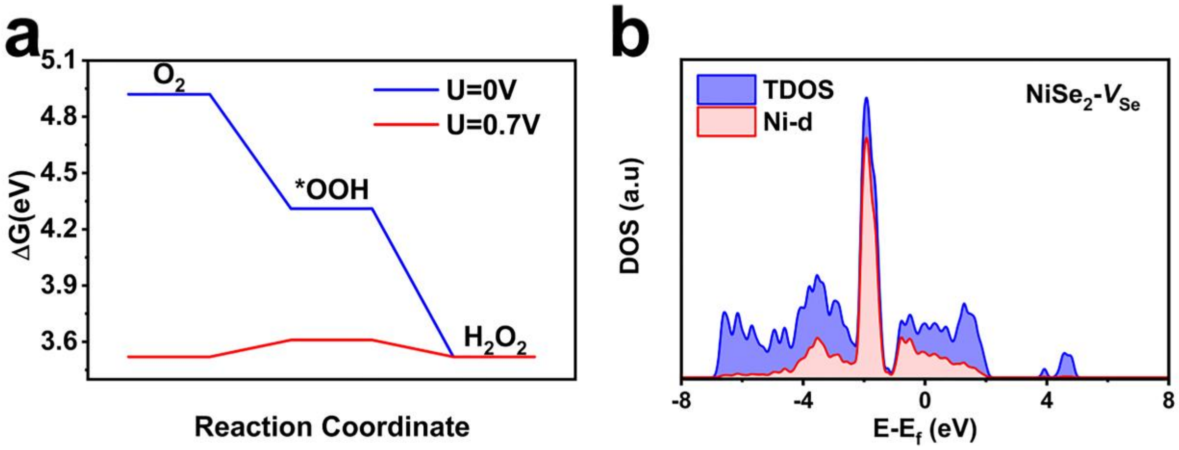


**Figure S28**. (a) Free energy diagram for 2e⁻ ORR on $NiSe_2$-$V_{Se}$-N–OOH system at U = 0 V and U = 0.70 V. (b) The Ni metal *d*-band center for $NiSe_2$-$V_{Se}$-N phase ($E - E_F$; relative to the Fermi level).

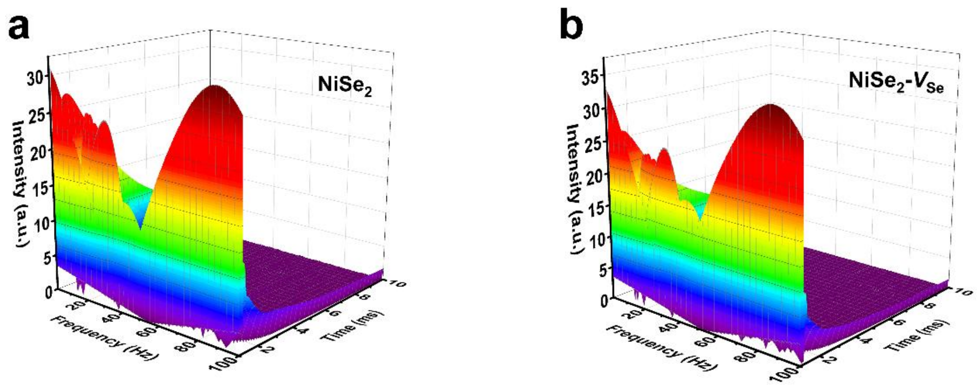


**Figure S29**. The continuous wavelet transformation (CWT) patterns of (a) $NiSe_2$ and (b) $NiSe_2$-$V_{Se}$ derived from the associated TPV curves.

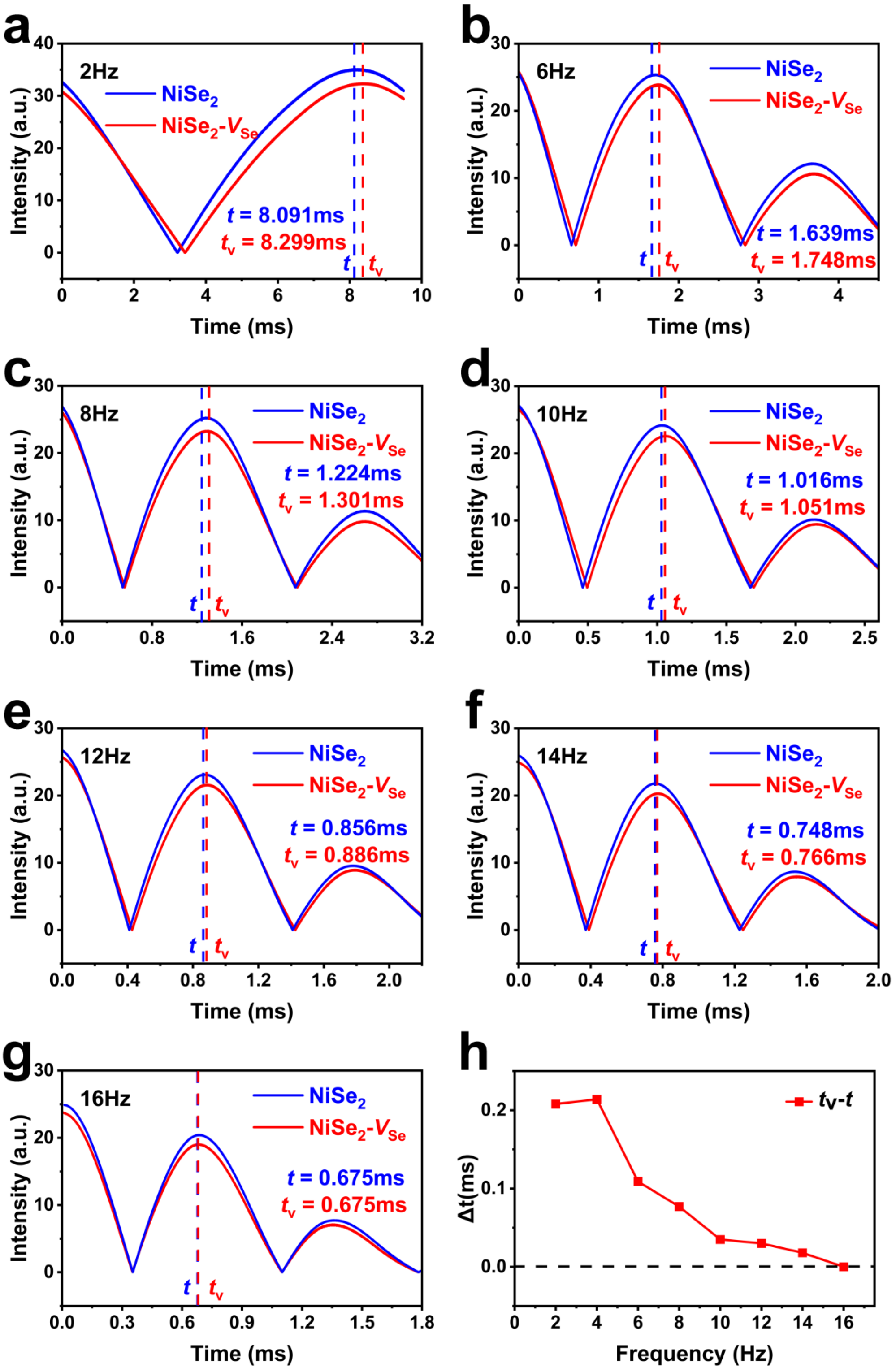


**Figure S30**. Comparison of intensity-time curves of $NiSe_2$ and $NiSe_2$-$V_{Se}$ under different frequencies, including (a) 2 Hz, (b) 6 Hz, (c) 8 Hz, (d) 10 Hz, (e) 12 Hz, (f) 14 Hz, and (g) 16 Hz. (h) Peak time difference ($\Delta t$) at different frequencies (f = 4, 6, 8, 10, 12, 14, and 16 Hz) for NiSe2 and $NiSe_2$-$V_{Se}$.

**TABLES**

**Table S1.** Structural parameters for $NiSe_2$ compounds before and after annealing under different temperatures refined from the experimental XRD profiles.

| Sample | Space group | Lattice parameters (Å) | | | Abundance (wt.%) |
|---|---|---|---|---|---|
| | | a | b | c | |
| $NiSe_2$ | $Pa\bar{3}$ | 5.9573(4) | 5.9573(4) | 5.9573(4) | 100 |
| $NiSe_2$-$V_{Se}$ | $Pa\bar{3}$ | 5.9663(2) | 5.9663(2) | 5.9663(2) | 100 |
| $NiSe_2$-$V_{Se}$-250 | $Pa\bar{3}$ | 5.9593(5) | 5.9593(5) | 5.9593(5) | 100 |
| $NiSe_2$-$V_{Se}$-300 | $Pa\bar{3}$ | 5.9614(5) | 5.9614(5) | 5.9614(5) | 100 |
| $NiSe_2$-$V_{Se}$-400 | $Pa\bar{3}$ | 5.9685(7) | 5.9685(7) | 5.9685(7) | 100 |
| $NiSe_2$-$V_{Se}$-450 | $Pa\bar{3}$ | 5.9715(9) | 5.9715(9) | 5.9715(9) | 100 |

**Table S2.** The comparison of $2e^-$ ORR performance of $NiSe_2$-$V_{Se}$ with those of recently reported Ni-based and other transition metal-based electrocatalysts in alkaline solution.

| Material | Electrolyte | Selectivity | Stability | Ref. |
|---|---|---|---|---|
| Ni–SA/G–0 | 0.1M KOH | ~94 | 3h | [6] |
| Mo SAC | 0.1M KOH | ~95 | 8h | [7] |
| Fe–CNT | 0.1M KOH | ~95 | 8h | [8] |
| Co–CNT | 0.1M KOH | ~75 | \ | [8] |
| Au-Pt-Ni | 0.1M KOH | ~91 | 10h | [9] |
| Co-POC | 0.1M KOH | ~83 | 10h | [10] |
| $Cu_{7.2}Se_4$ | 0.1M KOH | ~90 | \ | [11] |
| ZnS@C | 0.1M KOH | ~90 | 15h | [12] |
| $Fe_3O_4$/rGO | 0.1M KOH | ~63 | 5.5h | [13] |
| Cu-OC | 0.1M KOH | ~82 | \ | [14] |
| $Ba_2In_{1.75}Cu_{0.25}O_5$ | 0.1M KOH | ~80 | \ | [15] |
| $CuO_x$/rGO | 0.1M KOH | ~85 | 8.3h | [16] |
| $TiO_2$/TiC | 0.1M KOH | ~90 | 12h | [17] |
| N-$NiCo_2S_4$ | 0.1M KOH | ~89 | \ | [18] |
| Mn–O/N@NCs-20 | 0.1M KOH | ~70 | 4h | [19] |
| Mn-$TiO_2$ | 0.1M KOH | ~80 | 1.7h | [20] |
| $Ni_3(HITP)_2$ | 0.1M KOH | ~63 | \ | [21] |
| $NiSe_2$-$V_{Se}$ | 0.1M KOH | ~96 | 11h | This work |

**Table S3.** The peak time difference (Δt = $t_v$ – t) of $NiSe_2$ and $NiSe_2$-$V_{Se}$ at different frequencies.

| Frequency (Hz) | *t* (ms) | $t_v$ (ms) | Δ*t* = $t_v$-*t* (ms) |
|---|---|---|---|
| 2 | 8.091 | 8.299 | 0.208 |
| 4 | 2.610 | 2.824 | 0.214 |
| 6 | 1.639 | 1.748 | 0.109 |
| 8 | 1.224 | 1.301 | 0.077 |
| 10 | 1.016 | 1.051 | 0.035 |
| 12 | 0.856 | 0.886 | 0.030 |
| 14 | 0.748 | 0.766 | 0.018 |
| 16 | 0.675 | 0.675 | 0 |

**Reference**


[1] G. Kresse, D. Joubert, *Phys. Rev. B* **1999**, *59*, 1758.

[2] G. Kresse, J. Furthmüller, *Phys. Rev. B* **1996**, *54*, 11169.

[3] S. Chen, T. Luo, K. Chen, Y. Lin, J. Fu, K. Liu, C. Cai, Q. Wang, H. Li, X. Li, *Angew. Chem., Int. Ed.* **2021**, *133*, 16743.

[4] Z. Zhou, Y. Kong, H. Tan, Q. Huang, C. Wang, Z. Pei, H. Wang, Y. Liu, Y. Wang, S. Li, *Adv. Mater.* **2022**, *34*, 2106541.

[5] Y. Li, P. Peng, Z. Liao, F. Huo, Y. Liu, X. Shao, Z. Xiang, *ACS Sustainable Chem. Eng.* **2020**, *8*, 3728.

[6] X. Song, N. Li, H. Zhang, L. Wang, Y. Yan, H. Wang, L. Wang, Z. Bian, *ACS Appl. Mater. Interfaces* **2020**, *12*, 17519.

[7] C. Tang, Y. Jiao, B. Shi, J. N. Liu, Z. Xie, X. Chen, Q. Zhang, S. Z. Qiao, *Angew. Chem., Int. Ed.* **2020**, *132*, 9256.

[8] K. Jiang, S. Back, A. J. Akey, C. Xia, Y. Hu, W. Liang, D. Schaak, E. Stavitski, J. K. Nørskov, S. Siahrostami, *Nat. Commun.* **2019**, *10*, 1.

[9] Z. Zheng, Y. H. Ng, D. W. Wang, R. Amal, *Adv. Mater.* **2016**, *28*, 9949.

[10] B. Q. Li, C. X. Zhao, J. N. Liu, Q. Zhang, *Adv. Mater.* **2019**, *31*, 1808173.

[11] Q. Yuan, J. Zhao, D. H. Mok, Z. Zheng, Y. Ye, C. Liang, L. Zhou, S. Back, K. Jiang, *Nano Lett.* **2021**, *22*, 1257.

[12] C. Dai, R. Li, H. Guo, S. Liang, H. Shen, T. Thomas, M. Yang, *Dalton Trans.* **2021**, *50*, 5416.

[13] W. R. Barros, Q. Wei, G. Zhang, S. Sun, M. R. Lanza, A. C. Tavares, *Electrochim. Acta* **2015**, *162*, 263.

[14] F. He, Y. Zheng, H. Fan, D. Ma, Q. Chen, T. Wei, W. Wu, D. Wu, X. Hu, *ACS Appl. Mater. Interfaces* **2020**, *12*, 4833.

[15] S. Thundiyil, A. Pandikassala, S. Kurungot, R. N. Devi, *ChemElectroChem* **2022**, *9*, e202101163.

[16] H. Xiao, B. Li, M. Zhao, Y. Li, T. Hu, J. Jia, H. Wu, *Chem. Commun.* **2021**, *57*, 4118.

[17] Z. Xu, J. Liang, Y. Wang, K. Dong, X. Shi, Q. Liu, Y. Luo, T. Li, Y. Jia, A. M. Asiri, *ACS Appl. Mater. Interfaces* **2021**, *13*, 33182.

[18] X. Xu, X. Yin, J. Fu, D. Ke, *Chemistry* **2021**, *27*, 14451.

[19] A. Byeon, J. Cho, J. M. Kim, K. H. Chae, H.-Y. Park, S. W. Hong, H. C. Ham, S. W. Lee, K. R. Yoon, J. Y. Kim, *Nanoscale Horiz.* **2020**, *5*, 832.

[20] Q. Chen, C. Ma, S. Yan, J. Liang, K. Dong, Y. Luo, Q. Liu, T. Li, Y. Wang, L. Yue, *ACS*

*Appl. Mater. Interfaces* **2021**, *13*, 46659.

[21] E. M. Miner, T. Fukushima, D. Sheberla, L. Sun, Y. Surendranath, M. Dincă, *Nat. Commun.* **2016**, *7*, 1.
